# U^4+^ Speciation in Acidic Aqueous Solution: Insights from UV–Vis, EXAFS, XANES, and Quantum-Statistical Simulations

**DOI:** 10.1021/acs.inorgchem.5c01854

**Published:** 2025-06-16

**Authors:** Gema Raposo-Hernández, Rafael R. Pappalardo, Florent Réal, Valérie Vallet, Enrique Sánchez Marcos

**Affiliations:** † Department of Physical Chemistry, University of Seville, Seville 41012, Spain; ‡ Université de Lille, CNRS, UMR 8523-PhLAM, Physique des Lasers, Atomes et Molecules, Lille F-59000, France

## Abstract

This theoretical study investigates the UV–vis absorption properties of U^4+^-containing aqueous solutions and their relationship with the nature of aqua-complexes present at varying acidic levels. High-level quantum-mechanical calculations—accounting for relativistic effects, spin–orbit coupling, and both dynamic and nondynamic correlation—were combined with classical Molecular Dynamics simulations. EXAFS, XANES, and UV–vis spectra of U^4+^-containing aqueous solutions were used as experimental reference data and compared with the corresponding theoretical predictions. UV–vis spectra were available at various pH values. Theoretical spectra were generated as averages of individual spectra computed from the structures statistically generated. Coordination numbers ranging from 8 to 10 for the aqua ion were explored. Although the theoretical-experimental comparison of the EXAFS and XANES spectra allows us to reject ten-coordination, assigning the octa- or nine- (ennea-)­coordination to the U^4+^ aqua ion is difficult. However, UV–vis spectroscopy provided some evidence supporting a preference for the ennea-coordination. Spectra for aqueous solutions up to pH 2.22 were compared with simulated spectra of hydrolyzed forms of the aqua ion, in which up to two water molecules were replaced by hydroxyl anions. Spectra obtained as simulated mixtures of the aqua ion and hydrolyzed species in varying ratios produced a spectral evolution with pH that closely resembles experimental observations.

## Graphical abstract

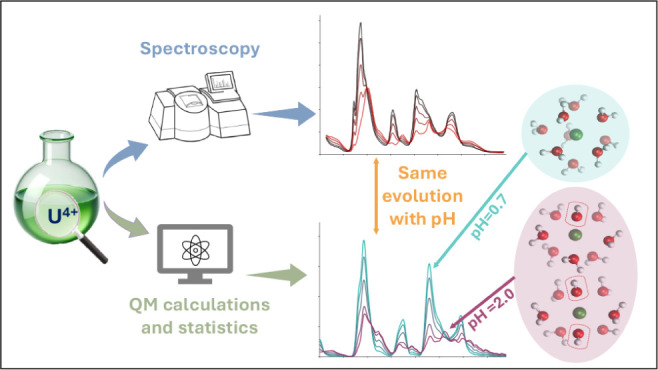

## Introduction

While not the most common oxidation state of uranium, the tetravalent ion U^4+^ predominates in strongly reducing conditions, such as those found in nuclear waste repositories. Although trivalent ions of the early actinoid series have been extensively studied and characterized, both as free ions and in solution, due to their importance in nuclear waste management and separation from the trivalent lanthanoids^1–8^ the tetravalent state has not received the same level of attention. A key challenge in nuclear waste management is the extraction and control of uranium and lanthanoid-containing species, which hinges on the solubility and mobility of their ions in different oxidation states under environmental conditions.^9,10^ Although numerous studies have addressed the hydrolysis of U^4+^ ^10–13^ a full understanding of its aqueous speciation remains elusive. Due to their high charge, tetravalent actinoids have a strong tendency to hydrolyze and polymerize, even at very low pH^11,14^ often resulting in the precipitation of oxides and hydroxides.^12^ However, the exact speciation as a function of pH is still under debate, and thermodynamic data vary depending on the experimental conditions.^13,15^

Spectroscopy offers valuable tools for identifying species in solution, especially when the electronic structure is sensitive to the surrounding environment. Techniques such as UV–vis, fluorescence, or X-ray absorption spectroscopy can provide critical insights into oxidation states, coordination environments, and molecular interactions.

The electronic structure of U^4+^ has been extensively investigated in crystalline metal hosts.^16–23^ The different chemical environments of uranium in these experiments have resulted in different splitting of its electronic states, which can be analyzed by crystal-field models. In both lanthanoids and actinoids, the f-orbitals are considered internal orbitals, with their electron occupation being responsible for optical activity. Their internal character implies that f-f transitions are only weakly influenced by the ligand field, allowing for meaningful connections between the energy levels of complexes and solvated ions with the corresponding gaseous free atoms or ions.^24^ Thus, studies of bare U^4+^ transitions have helped interpret more complex solid-state spectra. In addition to the valuable experimental data, theoretical studies on the electronic structure of the 5f^2^ manifold of U^4+^, including bare and solvation models^25–27^ as well as complexes with chloride^28^ or as an ionic impurity in crystals^29^ have been conducted. These studies have demonstrated the capacity of quantum mechanical (QM) methods to describe the spectra of static systems with reasonable accuracy, provided that static and dynamic electronic correlation as well as relativistic effects in the 5f shell are carefully treated.

However, the complexity increases in solution, where coordination numbers and ligand–metal distances fluctuate dynamically due to thermal motion and pH variations. In such cases, extended X-ray absorption fine structure (EXAFS) spectroscopy is a particularly useful tool for characterizing ions in solution, as it offers detailed local structural information and can be applied at low absorber concentrations.^30–33^ Although $U-O_{H_{2}O}$ bond distances derived from EXAFS fittings of U^4+^ solutions at low pH are generally consistent—typically around 2.40Å to 2.41Å—the coordination number remains uncertain, with reported values ranging from 8.7 to 10.5.^34–37^

To reduce this ambiguity, several studies have combined theoretical EXAFS spectrum simulations with statistical sampling of heavy-metal cation configurations.^33,38–43^ This strategy provides an advantage over purely experimental EXAFS fittings, where the uncertainty in coordination number is typically ± 1. Additionally, pure theoretical studies on the hydration dynamics of the ion in solution have been performed using QM/MM approaches^10,44^ as well as classical molecular dynamics simulations with polarizable force-fields^45^ to extract dynamical and structural information which can be confronted to experimental approaches.

Further experimental investigations into U^4+^ speciation across pH have shown that the stable pH range for the aqua ion is quite narrow.^13,46^ Like other tetravalent actinides, U^4+^ undergoes rapid hydrolysis, suggesting that even in acidic media, absorption spectra likely reflect contributions from both the pure aqua ion and its hydrolyzed forms.^14^ As a result, the exact speciation in acidic solutions remains unsolved, with divergent interpretations persisting in the literature,^12,13,15^

To address these challenges, we adopt a combined approach that integrates statistical configurational sampling with high-level quantum mechanical calculations. Previously, we applied this strategy to model the UV–vis spectra of the trivalent cerium Ce^3+^ ion in solution^47^ showing that both hydration number and water arrangement significantly influence spectral features.

In this work, we extend this methodology to the more involved case of tetravalent uranium, focusing on the UV–vis spectra of its predominant species at low pH, namely, the aqua ion ${\left[U{\left(H_{2}O\right)}_{n}\right]}^{4+}$ , along with its hydrolyzed forms ${[U{\left(\mathrm{OH}\right)}_{m}{\left(H_{2}O\right)}_{n}]}^{4-m}$ (*m* ≤ 2) suspected to be present up to pH≈ 2. This enables us to examine how coordination numbers and pH modulate the resulting spectral shapes. To complement the UV–vis analysis, we also simulate EXAFS and XANES spectra for these candidate species present in acidic aqueous solution, providing a more complete picture of U^4+^ speciation.

## Methodology

### QM Calculations

The computation of the electronic structure of bare U^4+^ and the hydrated ( ${\left[U{\left(H_{2}O\right)}_{n}\right]}^{4+}$ (*n* = 8,9,10)) or hydrolyzed ions ( ${[U{\left(\mathrm{OH}\right)}_{m}{\left(H_{2}O\right)}_{n}]}^{4-m}$ (*m* ≤ 2) (*n* = 6,7)), i.e., probing the f^2^ manifolds, three methods are explored, referred as **M1, M2** and **M3**. The initial level of theory chosen, **M1**, involves scalar relativistic all-electron calculations using the second-order Douglas-Kroll Hess (DKH) Hamiltonian.^48–50^ This approach employs all-electron atomic natural orbitals relativistic core correlation basis sets (ANO-RCC) with triple-ζ quality (ANO-RCC-VTZP) for O^51^ and H^52^ and quadruple-ζ quality (ANO-RCC-VQZP) for U.^53^ The ground and excited states wave functions were derived from state-average Complete Active Space Self Consistent Field (CASSCF)^54–56^ calculations, including two electrons distributed over the seven 5f orbitals, resulting in 21 triplet states and 28 singlet states. Dynamical correlation was incorporated using the second order perturbation theory CASPT2 ^57–59^ using the ionization potential electron affinity (IPEA) corrected zeroth-order Hamiltonian^60^ and keeping the 1s oxygen atomic orbitals, and all uranium core orbitals below the 5d orbitals frozen. An imaginary shift of 0.1*E*_h_ was used to avoid the presence of intruder states. The spin–orbit (SO) coupling was calculated using the state-interaction method (RASSI)^61^ using the atomic mean field (AMFI) SO integrals.^62,63^ All calculations were performed using OpenMolcas software.^64^

The second method employed, **M2**, introduces a change in the relativistic Hamiltonian. The literature indicates that the Exact Two-Component Hamiltonian (X2C)^65–69^ provides an accurate description of the relativistic effects on actinoids. Thus, this method utilizes the X2C Hamiltonian in conjunction with the X2C-TZVPall basis sets^70,71^ for O and H and the cc-pVQZ-X2C basis set^72^ for U. The adjustment of basis sets in **M2** is intended to enhance computational efficiency without compromising accuracy compared to experimental data. This is evidenced by the comparison of basis sets presented in Table S1 for the computation of vertical excitation energies for bare U^4+^. **M2’** refers to calculations using X2C with the ANO-RCC basis sets of **M1**, which results are quite close to those obtained by the **M2** method.

The third method **M3** retains the basis sets and the Hamiltonian used in **M2**, but it considers a larger orbital active space, that includes in addition to the seven 5f orbitals, the five 6d orbitals, as suggested by the work of Youshi et al.^73^ The number of roots are selected considering that we are interested in describing the f-f transitions, so we discard the 6d^2^ states, resulting in 56 triplet states and 63 singlet states, corresponding to the 5f^2^ and 5f^1^6d^1^ states. To verify the consistency of this procedure, we conducted the same calculations while including the 6d^2^ states (63 triplets and 78 singlets) to confirm the existence of an energy gap between the 5f^1^6d^1^ states and the 6d^2^ ones, that becomes around 3 eV. Furthermore, for the hydrolyzed species, it was necessary to extend the active space up to 13 orbitals, as the inclusion of the OH^–^ ligands in the U^4+^ complexes appears to stabilize the 7s orbital. Similarly to the approach adopted with the aqua ions, the states that are not required as part of the state average (6d^2^, 7s^2^, 7s6d) were discarded, necessitating the computation of 63 triplets and 70 singlets.

The computations for **M2** and **M3** including the spin–orbit (SO) coupling using mean-field approximation (SOMF)^74^ combined with the resolution of the identity (RI-SOMF­(1X)) were conducted using the ORCA 6.0 software.^75,76^

Optimizations and frequency calculations, which were subsequently used for Wigner sampling at room temperature (300 K), were also conducted with the ORCA software^75,76^ using density functional theory (DFT) with the PBE0 functional.^77^ The basis sets used are SD­(60,MWB)/DEF-TZVP^78–80^ for U and aug-cc-pVTZ^81^ for O and H. Solvation effects beyond the first coordination shell were accounted for using the CPCM solvation model.^82^

### Statistical Sampling: Molecular Dynamics and Wigner Distribution

In the context of U^4+^ UV–vis spectroscopy, the relevant excited states exhibit low absorption intensities, as they correspond to Laporte-forbidden f-f transitions. Consequently, it is essential to sample distorted molecular configurations to capture the intensity enhancements that arise from symmetry breaking and ligand-field splitting. Two complementary sampling methods were used to generate or to extract statistically significant sets of geometry for spectrum generation: Wigner sampling and classical Molecular Dynamics (MD) simulations. Classical MD simulations are a valuable technique for statistical sampling, especially when the force field (FF) parameters are derived from high-level quantum mechanical (QM) calculations. However, this approach presents notable limitations, including the time-consuming nature of FF parametrization and the requirement for ligand-specific parameter sets, which restricts its transferability. Acher et al.^45^ developed a set of FFs specifically tailored for describing tetravalent actinoid cations in water. A series of snapshots were extracted from 10 ns simulation of a cubic box containing the actinoid and 1000 water molecules. The water model used is TCPE/2013 ^83^ and a time step of 1 fs was appropriate due to the constrained geometry of the water molecule model. Analysis of the U–O radial distribution function (RDF) revealed a coordination number of 9, with the first solvation shell peak centered at 2.42 Å. Additional methodological details of the MD simulation can be found in reference.^45^

Another widely used technique for generated distorted molecular geometries is Wigner sampling^84–86^ which creates an ensemble of structures based on the harmonic normal vibrational modes obtained at the potential energy surface minimum of the target system.^87^ This method circumvents the need for classical FFs or computationally demanding ab initio molecular dynamics. However, it relies on the harmonic description, which can be less accurate for systems with low-frequency modes or high conformational flexibility. Despite this limitation, Wigner sampling is particularly well-suited for studying the complexation and speciation of heavy metals in aqueous environments, where metal–ligand interactions are predominantly ionic and the degree of anharmonicity is minimal. In this work, Wigner distributions were generated at 300 K based on the optimized structures and harmonic vibrational frequencies, using the *wigner.py* utility from the SHARC package.^88^

### Methodological Approach to Calculating Average XAS (EXAFS and XANES) and UV–Vis

The average EXAFS spectra were computed from 500 individual spectra obtained from sampled configurations. For the octa- and deca-hydrate in MD sampling, only 150 and 100 structures were extracted, respectively, due to their statistical representation over the 10 ns simulation. U L_III_-edge k^3^-weighted spectra were computed including multiple scattering up to four-legs and a maximum path length of 6Å. Details regarding the generation of EXAFS using self-consistent calculation of the wave function, with Heding-Lundqvist exchange correlation functional, can be found in prior works,^38,89,90^ An example of the FEFF input file to compute the EXAFS signal is provided in the Supporting Information.

To optimally match the simulated spectra with experimental data, the amplitude reduction factor, $S_{0}^{2}$ , and the energy correction of the threshold, Δ*E*_0_, are selected to make the first oscillation of the theoretical spectra matches the first oscillation of the different experimental ones. The values of Δ*E*_0_ are collected in Table S2. For the XANES spectra calculations, the number of individual spectra averaged was reduced to 100 sampled configurations, as this part of the XAS spectrum is less sensitive to geometrical fluctuations. In the SCF computations, the Heding-Lundqvist exchange correlation functional provided an ideal balance between the intensity of the white line and subsequent resonances. A shift of 5 eV in energy was applied to the theoretical spectra to match the experimental edge position. An input file for the XANES computation is given in the Supporting Information. EXAFS and XANES spectra have been simulated using the structural information and the theoretical scattering phases and amplitude functions computed by the ab initio FEFF code (v.9.6).^91^

The average UV–vis spectra were generated from 70 individual spectra represented as a sum of Gaussian functions centered on the transition energies and with an intensity corresponding to the oscillator strength. The full width at half-maximum was set to 0.015 cm^–1^. Convergence of the average UV–vis spectrum with the number of snapshots is shown in Figure S1.

For the specific case of the hydrolyzed species, 40 individual spectra have been used to reconstruct the final spectrum due to the difficulties in the AS selection. Despite this, 40 spectra were deemed sufficient to provide a representative picture of the main features and their relative intensities in the UV–vis spectrum.

## Results

### EXAFS Spectra

Table 1 presents the average distances of the first-shell water molecules for ${\left[U{\left(H_{2}O\right)}_{n}\right]}^{4+}$ with *n* = 8–10 derived from MD simulations, as well as their corresponding Debye–Waller factors (σ^2^). Additionally, the table includes data for (*n* = 8) and (*n* = 9) obtained from the Wigner sampling at 300 K. Notably, ${[U{\left(H_{2}O\right)}_{10}]}^{4+}$ is excluded from the Wigner distribution, as the optimization of the 10-water cluster resulted in a 9 + 1 structure, i.e., with one water molecule in the second coordination sphere. The final columns show the results of the fits to the experimental EXAFS spectra. In Figure 1, we compare the EXAFS spectra simulated for coordination 8 and 9 using MD sampling with the experimental ones available in the literature. An equivalent representation for the Wigner distribution is provided in Figure S2. Despite a noticeable difference of 0.04Å between the mean U–O distances of MD_8w and MD_9w—particularly evident at high *k*—both spectra align well with the experimental data. The relatively simple oscillation patterns of the spectra make it challenging to determine which one more closely resembles the experimental results. Moreover, while the intensity of the MD_9w spectrum is slightly greater, this increase is insufficient for a clear assignment of the U^4+^ coordination number when comparing the theoretical EXAFS results with the experimental ones.

**Table 1:** Comparison of the Computed U–O Average Distances (Å) and Debye–Waller (DW) Factors (Å^2^) with the Experimental Values Reported in the Literature.^34–37^

	MD		Wigner		Exp		
CN	R_U–O_	σ^2^	R_U–O_	σ^2^	R_U–O_	σ^2^	refs.
8	2.398	0.005	2.382	0.005			
9	2.439	0.007	2.415	0.005	2.41	0.007	ref.^35^
					2.40	0.009	ref.^37^
10	2.483	0.011			2.417	0.0084	ref.^34^
					2.40	0.0065	ref.^36^

**Figure 1:**
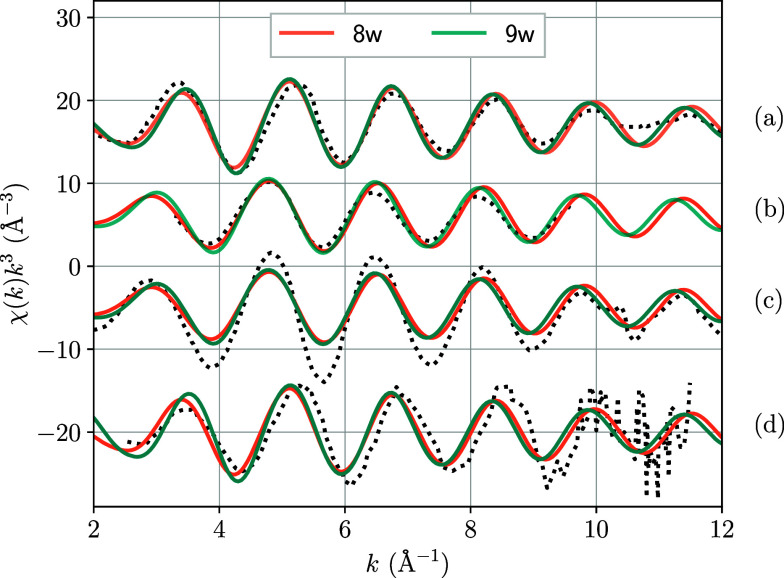
Average EXAFS spectra derived from MD sampling keeping structures holding either 8 (8w) or 9 (9w) water molecules. Dotted lines represent comparisons with the experimental spectrum from (a) Moll et al.,^34^ (b) Hennig et al.,^35^ (c) Ikeda-Ohno et al.^36^ and (d) A. Uehara et al.^37^ Note that the experimental spectra are presented with arbitrary intensity scaling.

The average EXAFS spectrum for ${[U{\left(H_{2}O\right)}_{10}]}^{4+}$ is included in Figure S3. A noticeable discrepancy in the oscillation frequency between the simulated and experimental spectra is observed, indicating that the structural model with a coordination number of 10 does not reproduce the experimental features accurately. Specifically, the average U–O bond distances in the simulated structure is up to 0.08Å longer than the value derived from the experimental fit.

In conclusion, there is a need for another spectroscopic technique to compare the results with and provide further insight into the discussion about the U^4+^ coordination in solution.

### XANES

An additional assessment using XAS spectroscopy involves comparing the average XANES spectra resulting from the aqua ions with the experimental one^36^ shown in Figure 2. An equivalent representation for the Wigner distribution has been included in Figure S4, showing that they are quite similar to those obtained using the MD snapshots. Similar to the EXAFS analysis, there is a reasonable agreement between the theoretical predictions and the experimental measurements. Notably, the two resonances above the white line are slightly better aligned for the ennea-coordination. However, the simulated spectra are not different enough to allow for a conclusive determination of the coordination number.

**Figure 2:**
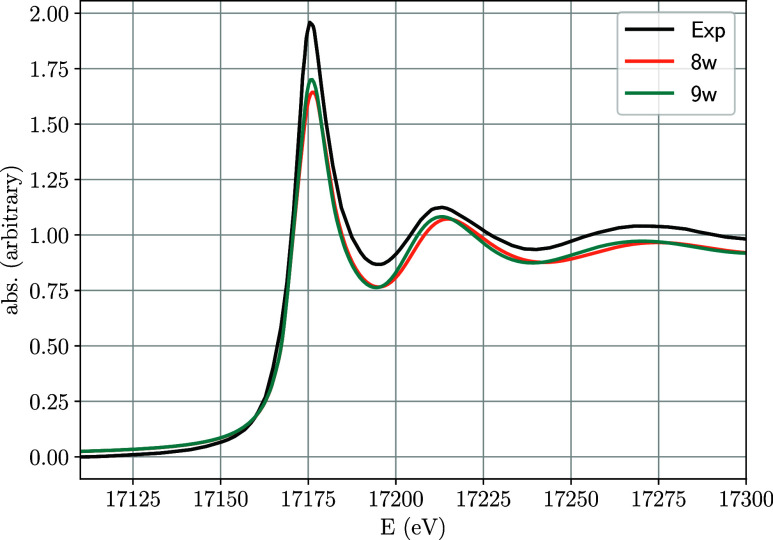
Comparison of the average XANES spectra from MD sampling keeping structures holding either 8 (8w) or 9 (9w) water molecules, with the experimental one recorded by Ikeda-Ohno et al.^36^.

### UV–Vis Spectra

#### U^4+^ Bare Spectrum

The relationship between the coordination number and the shape of the UV–vis absorption spectrum for 4f^1^ → 5d^1^ transitions was established in a previous study on Ce^3+^ ^47,92^ where the simulated spectra for the eight and nine coordinations were shown to be distinguishable. However, the electronic transitions for U^4+^ and its complexes observed in the visible range are f-f transitions. Due to the localized nature of the 5f orbitals, we can correlate the electron absorption spectrum of the bare U^4+^ with that of its aqua complexes.

In Table 2, we present a comparison of the experimental transition energies for U^4+^ ^93,94^ with the results of MRCI+DC calculations conducted by one of us^26^ and the CASPT2 results of this study. The last rows of the table list the Mean Absolute Error (MAE) with respect to the experimental value. The MRCI+DC computation^26^ serves as reference calculation, even though its overestimation of the highest state J = 0 (46 189 cm^–1^). To address this discrepancy, we included an additional row for the MAE, excluding the last transition, labeled MAE’. We observe that the deviations of the CASPT2 results increase with the transition energy values. In a study on bare U^4+^ spectroscopy, Barandiarán and Seijo^25^ applied a correction factor to the spin–orbit (SO) operator, yet the ^3^P and ^1^I related spin free states were still overestimated by about 1000 cm^–1^. Consequently, these energy levels were adjusted downward to better align the computed spin–orbit transition energies with experimental values. In our approach, we applied a reduction factor of 5% to M1 and M2 transitions and 7% to M3 transitions to achieve closer alignments with the experimental results. This adjustment reduced the resulting MAE by nearly half, yielding excellent agreement with experimental data. These findings support the applicability of our methodology to complexes, such as aqua ions and their hydrolyzed forms.

**Table 2:** Comparison of the so-Caspt2 Energy Values (cm^–1^) of f-f Transitions of Bare U^4+^ with the Experimental Results^a^

State		DKH			X2C			
			AS (2,7)				AS (2,12)	
J	Expt.^93,94^	MRCI+DC^26^	M1	M1*0.95	M2	M2*0.95	M3	M3*0.93
4	0	0	0	0	0		0	
2	4161	4501	4198	3988	3956	3758	4184	3891
5	6137	6392	6604	6274	6448	6126	6600	6138
3	8984	9455	9366	8897	8977	8528	9347	8693
4	9434	9819	9713	9228	9402	8931	9742	9060
6	11514	12010	12335	11718	12075	11471	12354	11489
2	16465	17531	17239	16377	16906	16061	17410	16191
4	16656	17289	17295	16430	17370	16502	17586	16355
0	17128	18170	17972	17073	18022	17121	18248	16970
1	19819	20960	21264	20200	21257	20195	21518	20012
6	22276	23744	24420	23199	24276	23062	24551	22832
2	24653	25998	26195	24885	26116	24811	26606	24744
0	43614	46189	43368	41199	43237	41075	44468	41355
MAE		935	802	427	704	487	981	399
MAE’		786	852	247	733	300	993	230

a The Mean Absolute Error (MAE) Have Been Computed. MAE’ Does Not Take into Account the Last Transition. Two Active Spaces (AS) Were Used, One with 2 Electrons in the 5f Orbitals (AS­(2,7)) and a Second One with 2 Electrons in the 5f and 6d Orbitals (AS­(2,13)). Scaling Factors of 0.95 and 0.93 Were Applied to Align the Calculated Results with the Experimental Data More Closely

#### Impact of the Coordination Number and Active Spaces on the Aqua Ion Transition Energies

The next step in our study is to assess how well the shape and position of the bands observed in the experimental UV–vis spectrum of tetravalent uranium in solution can be reproduced through simulations of average spectra. A primary objective is to look for potential correlations between the coordination number and the resulting position or shape of these bands. To this end, a set of representative geometrical configurations is used to compute the individual UV–vis spectra, which are then averaged and convoluted using Gaussian functions with a fixed half-width. Based on the data in Table 2, we anticipate that the three methods will yield comparable spectral trends when progressing from the bare U^4+^ ion to its hydrated forms.

While UV–vis spectra are commonly presented in terms of wavelength (nm), in this study we have chosen to report and discuss them in wavenumbers (cm^–1^). This choice is motivated by the fact that the more involved spectral region (from 14 000 cm^–1^ to 25 000 cm^–1^ or from 400 to 700 nm) is more intuitively analyzed when expressed in terms of energy. For completeness, the corresponding spectra in wavelength units are provided in the Supporting Information.

Figure 3a presents the first set of theoretical spectra for the U^4+^ aqua ion, obtained using method M1, specifically for configurations featuring eight, nine or ten water molecules in the first coordination shell as selected from MD trajectories. In parallel, Figure 3b shows the average spectra of the octa and ennea-hydrate, computed using their Wigner distributions. As already mentioned for the EXAFS case, the spectrum corresponding to the deca-hydrate cannot be computed since the geometrical optimization of ${[U{\left(H_{2}O\right)}_{10}]}^{4+}$ aqua ion leads to a 9 + 1 structure. All theoretical transition energies shown have been adjusted using the same scaling factors determined for the atomic case, as detailed in Table 2. For completeness, Figures S5 and S6 provide the spectra represented in wavelength and without normalization, respectively.

**Figure 3:**
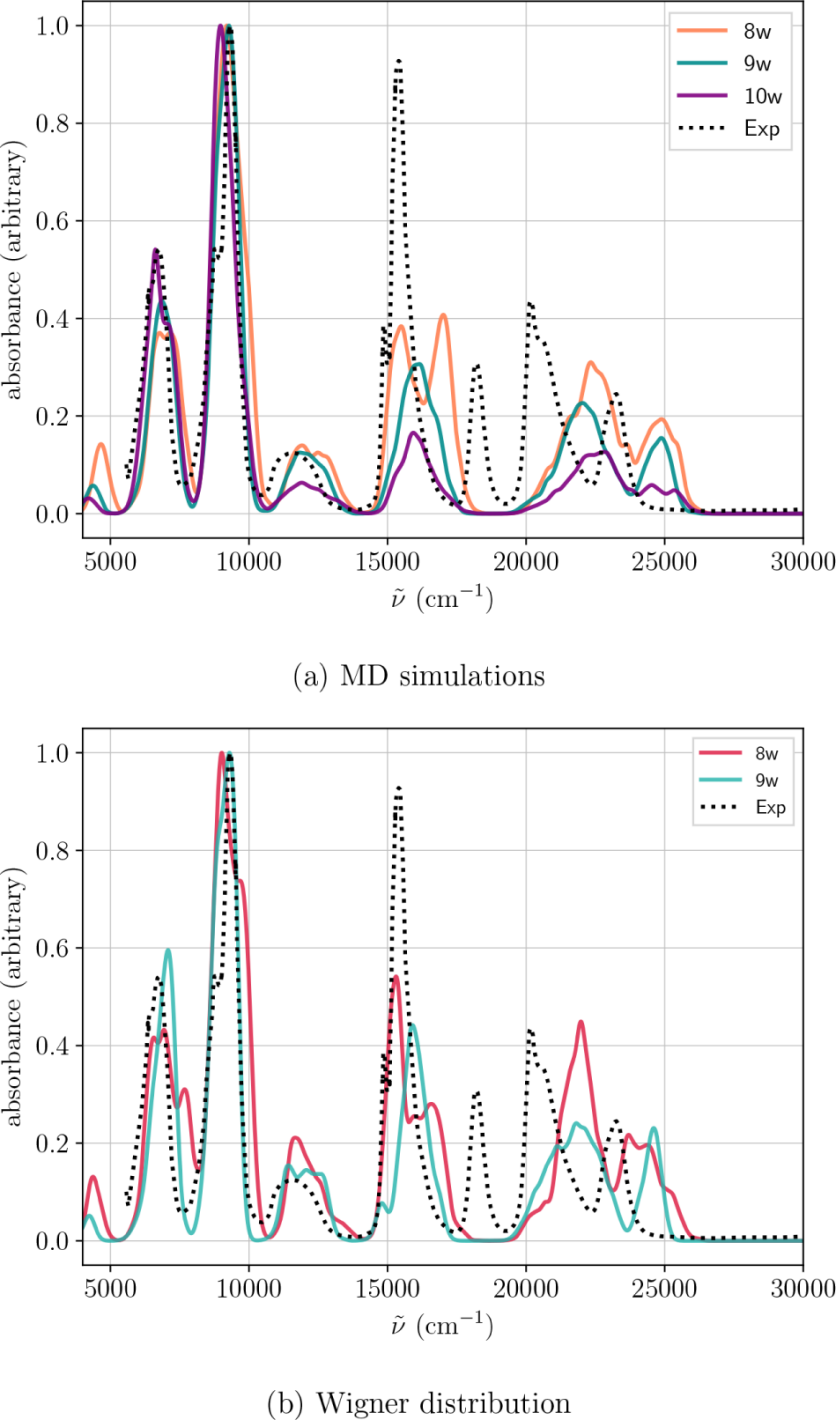
Average spectra for ${\left[U{\left(H_{2}O\right)}_{n}\right]}^{4+}$ (a) from MD simulation with *n* = 8, 9, 10, and (b) from Wigner distribution with *n* = 8, 9, calculated using the M1 method (7 orbitals in the AS).

From these results, two key conclusions can be drawn. First, the overall spectral shape is well reproduced, particularly in the low-energy region spanning 5000 cm^–1^ to 14 000 cm^–1^. Second, notable spectra differences emerge with varying coordination numbers. They are especially pronounced in the region between 15 000 cm^–1^ to 17 000 cm^–1^ and 20 000 cm^–1^ to 25 000 cm^–1^ of Figure 3, where an inverse correlation between intensity and coordination number becomes evident. However, the comparison of the simulated spectra of the octa- and ennea-hydrates with the experimental one is not clear enough to definitively determine the coordination number of the hydrated ion. Moreover, the spectral region between 20 000 cm^–1^ to 25 000 cm^–1^ is not well captured, with a particularly notable absence of the experimental transition at 18 000 cm^–1^. A similar observation was reported by Autillo et al.^95^ who performed SO-CASPT2 calculations on various U^4+^ aqua complexes with coordination numbers of 8, 9, and 10. Their spectra-shown at the bottom of Figure 7 of their study-display a comparable trend in the 15 000 cm^–1^ to 25 000 cm^–1^ region as the coordination number changes, and they also failed to reproduce the transition near 18 000 cm^–1^.

Given the close similarity between MD and Wigner averaged spectra, we focus exclusively on the results obtained via Wigner sampling in the subsequent discussion.

We now turn to the results obtained using the M2 method shown in Figure 4. A notable difference emerges in the high-energy region when switching from the relativistic DKH Hamiltonian (M1) to the X2C one (M2). In particular, the higher-energy bands in the M2 spectra exhibit a red shift relative to M1. Additionally, those new spectra show a very low-intensity peak at 19 000 cm^–1^ that could correspond to the experimental transition observed at 18 000 cm^–1^, which was absent in the spectra calculated by the M1 method. In contrast, the low-energy region of the spectra remains largely unchanged between the two methods. It is important to note that f-f transitions are parity forbidden in atoms, resulting in relatively small oscillator strengths in complexes, with a maximum value in the order of 10^–5^. These small intensities arise primarily due to ligand-field splitting and the breaking of atomic symmetry. Consequently, even minor modifications of the molecular wave functions can result in significant changes in the relative intensity of the bands of the theoretical spectra. Comparable M1/M2 differences were also observed in the spectra generated MD-based sampling, as shown in Figure S7. Figure S8 includes the same plots in wavelength.

**Figure 4:**
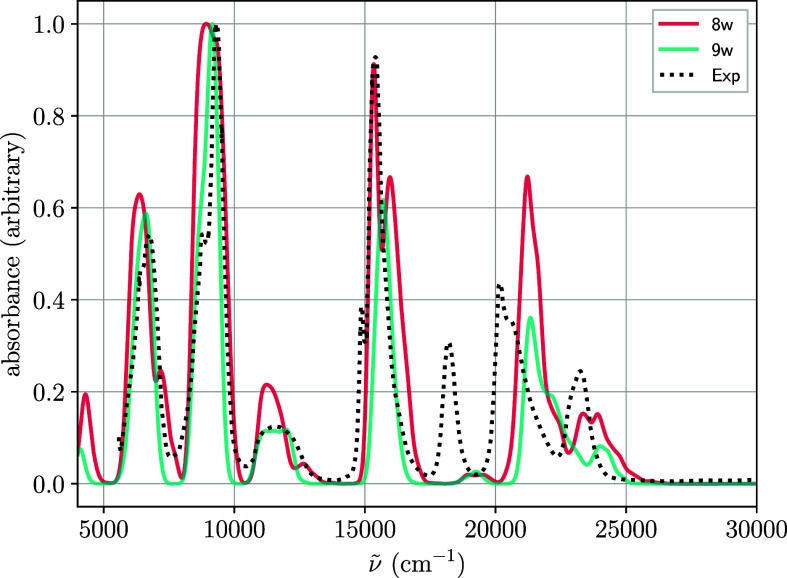
Average spectra from Wigner distribution for ${\left[U{\left(H_{2}O\right)}_{n}\right]}^{4+}$ with *n* = 8, 9 calculated using the M2 method (7 orbitals in the AS).

The final attempt to achieve a comprehensive description of the experimental spectrum involves extending the AS from 7 to 12 orbitals (method M3). The resulting spectra are shown in Figure 5. Although the 6d orbitals are not involved in the transitions under investigation, their inclusion in the active space is justified, as they contribute to angular correlation. Moreover, 5f^2^ → 5f^1^6d^1^ transitions occur at about 30 000 cm^–1^ higher than the f-f ones.^27,28^ and can indirectly affect the relative intensity distribution through state interaction.

**Figure 5:**
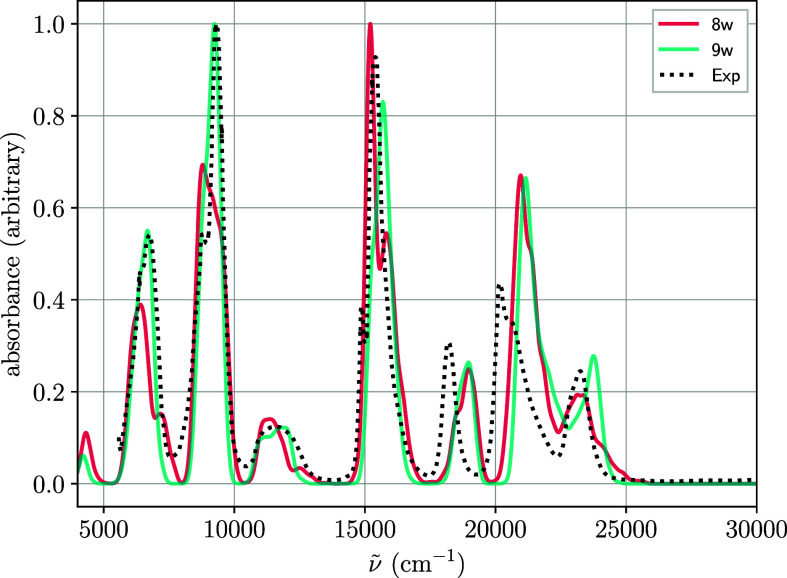
Average spectrum from Wigner distribution for ${\left[U{\left(H_{2}O\right)}_{n}\right]}^{4+}$ with *n* = 8, 9 calculated using the M3 method.

Notably, the relative intensity of the bands of the upper part of the spectra are enhanced compared to the M2 results. Thus, the experimental band at 18 000 cm^–1^, previously absent in M1 and only weakly present in M2, is now recovered, albeit with a blue shift of approximately 1000 cm^–1^ in the 18 000 cm^–1^ to 25 000 cm^–1^ region. This enhanced theoretical approach allows for a more detailed, band-by-band comparison with the experimental spectrum, thereby enabling a more informed evaluation of the coordination number. The relative intensities of the three most intense experimental bands (7000 cm^–1^, 9000 cm^–1^ and 16 000 cm^–1^) are more accurately represented by the ennea-hydrate spectrum (blue line in Figure 5). For the high-energy region (18 000 cm^–1^ to 25 000 cm^–1^) the spectra for the two coordinations are rather similar. The results obtained using MD sampling are also similar, as shown in Figure S9. Figure S10 includes the same plots in wavelength. The spectra derived from the MD simulations (see Figure S10a) lead to similar conclusions. The spectra shapes derived from the two coordinations are quite close. However, the spectra derived from the Wigner distributions (Figure S10b) show better similarity with the experimental spectrum for the ennea-coordination in the 1000 to 1700 nm region. Thus, we can conclude that both coordination geometries capture the main spectral features, making it difficult to unambiguously assign a preferred coordination based on a visual inspection of such involved spectroscopical functions. In any case, based on the slightly better performance of the ennea-coordination, we will focus on this hydrate for the remainder of the study.

#### Effect of pH on the Computed Spectra

Several authors have already reported that the pH range in which the U^4+^ aqua ion exists is extremely narrow, as the tetravalent actinides, including U^4+^, exhibit a strong tendency to hydrolyze. As a result, the absorption features observed in experimental measurements may arise from hydrolyzed species or from a mixture of these species with the unhydrolyzed aqua ion.^14^ As discussed in the Introduction, the speciation of U^4+^ in acidic media remains a topic of ongoing debate, with varying interpretations found across the literature.^12,13,15,96^ Nevertheless, there is a consensus that species containing more than two hydroxide ligands are unlikely to form at pH < 2. For this reason, the present study focuses exclusively on species with one or two OH^–^ groups.

Previous studies^97,98^ have shown that, for highly charged cations, the substitution of coordinated water molecules by hydroxide ligands upon deprotonation tends to reduce the overall coordination number. Taking into account the premise that the aquo ion has a CN of 9, it can be extrapolated that hydrolyzed species would exhibit lower CN values, in the range of 7–8. Based on this, we consider the following candidate species: ${[U\left(\mathrm{OH}\right){\left(H_{2}O\right)}_{6}]}^{3+}$ , ${[U\left(\mathrm{OH}\right){\left(H_{2}O\right)}_{7}]}^{3+}$ , ${[U{\left(\mathrm{OH}\right)}_{2}{\left(H_{2}O\right)}_{5}]}^{2+}$ and ${[U{\left(\mathrm{OH}\right)}_{2}{\left(H_{2}O\right)}_{6}]}^{2+}$ . Due to the lack of available force fields for hydrolyzed tetravalent actinides, statistical sampling for these species was performed exclusively via the Wigner method.

The UV–vis of the proposed hydrolyzed species, computed using the M3 method, along with their comparison to the experimental spectrum, are shown in Figure 6. It is worth pointing out that the spectral range accessible for these species is significantly narrower than that of the aqua ion, spanning from 12 000 cm^–1^ to 28 000 cm^–1^. As seen in the figure, none of the simulated spectra fully reproduces the features of the experimental spectrum. Among them, the monohydroxide species exhibit the closest resemblance in terms of overall spectral shape; however, the relative intensities of the bands are not accurately captured. The experimental spectrum used for comparison was obtained by deconvoluting the data from Cha et al.^99^ collected in the pH range 0.7 to 2.22. The authors assigned the observed bands to the UOH^3+^ complex (that is the reason we use this label in Figure 6). This discrepancy leads us to hypothesize that the species considered in our calculations may not fully represent the dominant species in solution under these conditions. Alternatively, the experimental spectrum may correspond to a mixture of species, rather than a single, well-defined uranium complex.

**Figure 6:**
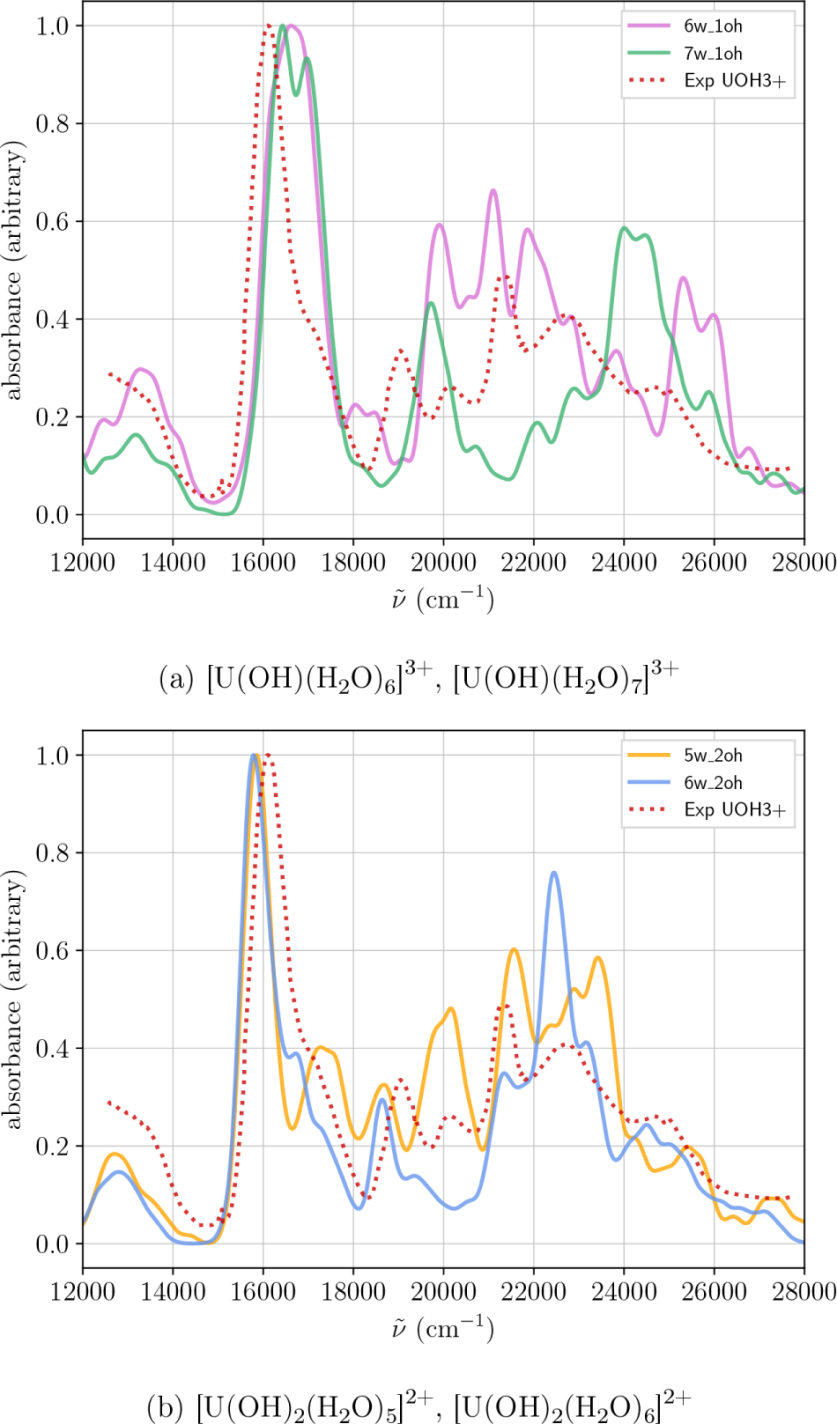
Average spectrum from Wigner distribution for (a) singly hydrolyzed species ${[U\left(\mathrm{OH}\right){\left(H_{2}O\right)}_{6}]}^{3+}$ and ${[U\left(\mathrm{OH}\right){\left(H_{2}O\right)}_{7}]}^{3+}$ and doubly hydrolyzed species (b) ${[U{\left(\mathrm{OH}\right)}_{2}{\left(H_{2}O\right)}_{5}]}^{2+}$ and ${[U{\left(\mathrm{OH}\right)}_{2}{\left(H_{2}O\right)}_{6}]}^{2+}$ calculated using the M3 method. Experimental data are taken from reference at pH = 2.^99^

In order to address the aforementioned uncertainty, we simulated the spectral evolution with increasing pH by using species ratios derived from speciation diagrams. This approach enables a direct comparison with the experimentally observed spectral evolution, and, in principle, allows us to identify the species present in solution at higher pH by evaluating which mixture best matches the experimental trends. The most recent thermodynamic data for the hydrolysis constants of U^4+^ were reported by Yan et al.^96^ and the corresponding speciation diagram based on these new constants is included in Figure S11. Using the species distributions extracted from this diagram, as well as from the earlier data by Lehmann et al.^13^ we reconstructed the evolution of the absorption spectra with pH. The applied ratios are summarized in Table S3. The four possible combinations of hydrolyzed species and their mixing are represented in Figure S12 for the constants reported in Yan et al.^96^ and in Figure S13 for the speciation diagram from ref.^13^ In both cases, the best agreement with the experimental spectra is achieved when considering species with a total CN = 8, namely, ${[U\left(\mathrm{OH}\right){\left(H_{2}O\right)}_{7}]}^{3+}$ and ${[U{\left(\mathrm{OH}\right)}_{2}{\left(H_{2}O\right)}_{6}]}^{2+}$ .

Figure 7 presents the comparison between the experimental UV–vis spectral evolution with pH and the corresponding simulated spectra based on the best-fitting species ratios using the most recent thermodynamic data. At first sight, the theoretical spectra reproduce the experimental trends remarkably well across the entire spectral range. Notably, the main spectral features—such as the strong band at 16 000 cm^–1^, the emerging shoulder at 17 000 cm^–1^ with increasing pH, the band at 19 000 cm^–1^ which nearly vanishes at pH = 2.0, and the decrease in intensity of the 21 000 cm^–1^ band—are all closely captured. Smaller variations in relative intensity are also well reproduced, particularly in the features observed around 20 000 cm^–1^, 23 000 cm^–1^ and 25 000 cm^–1^. The overall agreement is especially striking when comparing the theoretical and experimental spectra at pH = 2.0, where both the intensity ratios and band positions are nearly identical.

**Figure 7:**
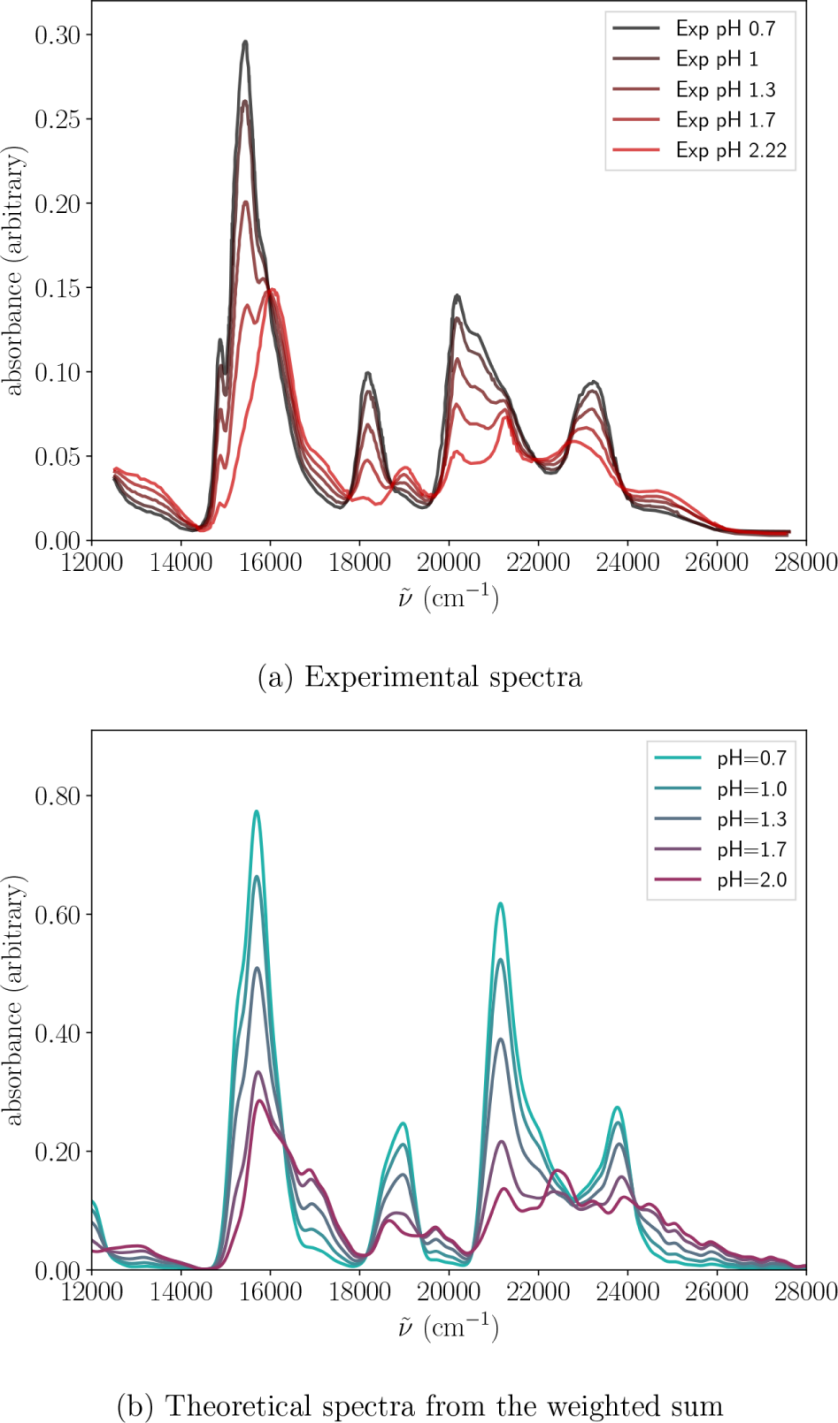
a) Experimental spectra measured by Cha et al.^99^ as a function of pH. b) Weighted sum of ${[U{\left(H_{2}O\right)}_{9}]}^{4+}$ , ${[U\left(\mathrm{OH}\right){\left(H_{2}O\right)}_{7}]}^{3+}$ and ${[U{\left(\mathrm{OH}\right)}_{2}{\left(H_{2}O\right)}_{6}]}^{2+}$ Wigner-generated spectra to show the effect of the percentage of hydrolysis. The percentages employed are summarized in Table S3, where the data is limited to pH = 2.0.

Figure S14 shows the set of simulated spectra at different pH values, assuming that the aqua ion was the octahydrate. In this case, the singly and doubly hydrolyzed species should be ${[U\left(\mathrm{OH}\right){\left(H_{2}O\right)}_{6}]}^{3+}$ and ${[U{\left(\mathrm{OH}\right)}_{2}{\left(H_{2}O\right)}_{5}]}^{2+}$ . Although the overall trend—red-shifting with increasing deprotonation-is captured, the spectral shapes in the 15 000 cm^–1^ to 17 000 cm^–1^ and 22 000 cm^–1^ to 26 000 cm^–1^ regions deviate significantly from experiment. By contrast, the mixed-species simulations incorporating the ennea-hydrate, ${[U{\left(H_{2}O\right)}_{9}]}^{4+}$ , reproduce these features far more faithfully (see Figure 7). This last result, along with our previous comparison, compels us to conclude that the ennea-hydrate could be the preferred species in acidic media. This confirms that advanced simulation techniques have reached a sufficient level of maturity to provide valuable insight into the speciation behavior of heavy elements.

## Concluding Remarks

This study has explored the coordination environment of the highly charged U^4+^ cation in aqueous solution by comparing experimental spectra from three techniques—EXAFS, XANES and UV–vis—with simulated spectra obtained by combining quantum and statistical methods for various ${\left[U{\left(\mathrm{OH}\right)}_{m}{\left(H_{2}O\right)}_{n}\right]\left(4-m\right)}^{+}$ complexes. At pH 0, the predominant species is the fully hydrated aqua ion. Simulated EXAFS and XANES spectra for both the octa- and ennea-hydrate complexes show good agreement with experimental spectra available in the literature, supporting the widely accepted perception that the XAS techniques typically estimate the coordination number with an uncertainty of ± 1. Notably, while this uncertainty is commonly attributed to the limitations of experimental EXAFS fitting, in this work, a similar uncertainty arose but from direct comparison between simulated spectra of species with two different coordination numbers.

UV–vis spectroscopy alone does not provide a definitive coordination value for the U^4+^ aqua ion. However, when combined with theoretical spectra generated from structures with known coordination environments, UV–vis analysis becomes a valuable tool for speciation. This approach enabled us to conclude that the ennea-coordination is the most likely structure for the U^4+^ fully hydrated aqua ion at low pH. However, due to the similar results obtained for the octa-aqua ion, this coordination cannot be definitively discarded.

A significant methodological contribution of this work—previously highlighted in our study on Ce^3+^ hydration^47^—is the application of Wigner sampling. This technique provides a statistically meaningful ensemble of structures around the equilibrium geometry using only the quantum-mechanical optimized structure and its vibrational frequencies. Wigner sampling yields spectral results comparable to those obtained from molecular dynamics simulations, but with a substantially lower computational cost.

Additionally, this work has extended the analysis to hydrolyzed species formed upon at higher pH values. Our results demonstrates that weighted spectra—constructed by combining contributions from the aqua ion and hydrolyzed species in ratios derived from speciation models—successfully reproduce the experimental UV–vis spectral evolution across the pH range. Among the candidate species, ${[U\left(\mathrm{OH}\right){\left(H_{2}O\right)}_{7}]}^{3+}$ and ${[U{\left(\mathrm{OH}\right)}_{2}{\left(H_{2}O\right)}_{6}]}^{2+}$ provide the best agreement with the experimental spectra, supporting their predominance in mildly acidic conditions.

## Supplementary Material



## Notes

OpenMOLCAS, ORCA FEFF input files. Table summarizing the computed transition energies of the bare U^4+^ (Table S1). Figure S1 illustrates the convergence of the computed spectra average as the number of snapshots increases. Figure S2, Figure S3 and Figure S4 present additional data on the computed EXAFS spectra. Figure S5, Figure S6, Figure S7, Figure S8, Figure S9, and Figure S10, present additional UV–vis spectra. Speciation diagram Figure S11 and corresponding ratio of the aqua ions and hydrolyzed species in Table S3. Computed spectra in Figure S12 and Figure S13 using ${[U{\left(H_{2}O\right)}_{9}]}^{4+}$ and the different ratios of the spectral contributions of the ${[U{\left(\mathrm{OH}\right)}_{m}{\left(H_{2}O\right)}_{n}]}^{4-m+}$ (*m* = 1, 2, *n* = 5–7). Figure S14 using ${[U{\left(H_{2}O\right)}_{8}]}^{4}$ and the different ratios of the spectral contributions of the ${[U\left(\mathrm{OH}\right){\left(H_{2}O\right)}_{6}]}^{3+}$ and ${[U{\left(\mathrm{OH}\right)}_{2}{\left(H_{2}O\right)}_{5}]}^{2+}$ ­(PDF)
