# Supplementary material for: U4+ Speciation in Acidic Aqueous Solution: Insights from UV–Vis, EXAFS, XANES, and Quantum-Statistical Simulations
Source: Inorg Chem. 2025 Jun 16;64(30):15321–31. doi: 10.1021/acs.inorgchem.5c01854 (PMC12326364; doi:10.1021/acs.inorgchem.5c01854)
Supplement: Supplementary file 1 [file ic5c01854_si_001.pdf]

Supporting Information: U^{4+} Speciation in Acidic Aqueous Solution: Insights from UV-Vis, EXAFS, XANES and Quantum-Statistical Simulations

Gema Raposo-Hernández,† Rafael R. Pappalardo,† Florent Réal,‡ Valérie Vallet,‡ and Enrique Sánchez Marcos*,†

†*Department of Physical Chemistry, University of Seville, 41012-Seville, Spain*

‡*Université de Lille, CNRS, UMR 8523-PhLAM, Physique des Lasers, Atomes et Molecules, F-59000 Lille, France*

E-mail: sanchez@us.es

Contents

1 OpenMolcas input **S-3**

2 ORCA input **S-5**

3 Excitation Energies of bare U^{4+} **S-7**

4 E_0 employed for the EXAFS computation **S-7**

5 Feff input for EXAFS **S-8**

6 Feff input for XANES **S-9**

7 Convergence of the average UV-vis spectra **S-11**

8 EXAFS and XANES spectra **S-12**

9 Average UV-vis spectra computed with M1 method in wavelength **S-14**

10 Average UV-vis spectra computed with M1 method without normalisation **S-15**

11 Average UV-vis spectra computed with M2 method **S-16**

12 Average UV-vis spectra computed with M2 method in wavelength **S-17**

13 Average UV-vis spectra computed with M3 method **S-18**

14 Average UV-vis spectra computed with M3 method in wavelength **S-19**

15 Speciation as a function of pH **S-20**

References **S-24**

1 OpenMolcas input

```
/*
 Define geometry, basis set and point group
 Start the calculation with a minimal basis set
 Coordinates from MD with 8w (149 in 10000 structures)
*/
 &GATEWAY

Coord = $CurrDir/x0113.xyz
 Group=C1                // Force no symmetry
 Basis set = U.ANO-RCC-VQZP, O.ANO-RCC-VTZP, H.ANO-RCC-VTZP

 Douglas-Kroll           // Douglas-Kroll Hamiltonian

 RICD

/*
  Compute integrals with Cholesky decomposition
*/
 &SEWARD

/*
 Perform a CASSCF calculation on singlet states (5f2)
*/
 &RASSCF
   Spin=1                     // Multiplicity
   Symmetry=1                 // Symmetry of the states
   Inactive=83                // Doubly occupied orbitals
   Ras2=7                     // Active orbitals (7 5f orbitals)
   Nactel=2 0 0               // Number of active electrons (2 5f electrons)
   CIRoots=28 28 1            // Number of singlet states with equal weights
>>  COPY $Project.JobIph $Project.JobIph.singlets

/*
  Perform a XMS-CASPT2 calculation for the singlet states
*/
  &CASPT2
    IPEA=0.25
    XMUL=ALL
    Frozen=47
    MaxIter= 80
>>  COPY $Project.JobMix $Project.JobMix.singlets

/*
```

```
 Perform a CASSCF calculation on triplets states (5f2)
*/
 &RASSCF
   Spin=3                    // Multiplicity
   Symmetry=1                // Symmetry of the states
   Inactive=83               // Doubly occupied orbitals
   Ras2=7                    // Active orbitals (7 5f orbitals)
   Nactel=2 0 0              // Number of active electrons (2 5f electrons)
   CIRoots=21 21 1           // Number of singlet states with equal weights
>>  COPY $Project.JobIph $Project.JobIph.triplets

/*
  Perform a XMS-CASPT2 calculation for the triplet states
*/
  &CASPT2
    IPEA=0.25
    XMUL=ALL
    Frozen=47
    MaxIter= 80
>>  COPY $Project.JobMix $Project.JobMix.triplets

/*
  Perform a SO-coupling calculation with the singlets and triplets CASPT2
*/
>> COPY $Project.JobMix.singlets JOB001
>> COPY $Project.JobMix.triplets JOB002
 &RASSI;
SPINorbit;
EJOB
OMEGa
End of Input
```

2 ORCA input

```
! SP x2c-TZVPall
! X2C RIJK
! CASPT2
! MOREAD
%moinp "x0001_9w_cpcm_scal_caspt2.gbw"
%maxcore  16000
%pal nprocs 26 end
%method
  FrozenCore -96
End
%casscf
 nel 2
 norb 12
 mult 3,1
 nroots 56,63
  PTSettings
  CASPT2_rshift 0.0
  CASPT2_ishift 0.1
  CASPT2_IPEAshift 0.25
  end
 rel
   dosoc true #spin-orbit coupling (and ZFS)
 end
end

#R. Feng and K.A. Peterson, J. Chem. Phys. 147, 084108 (2017).
#eXact 2-Component correlation consistent polarized Valence Triple-Z Basis Set for U
#!----------------------------------------------------------------------
#! Basis Set Exchange
#! Version 0.10
#! https://www.basissetexchange.org
#!----------------------------------------------------------------------
#!   Basis set: cc-pVQZ-X2C
#! Description: cc-pVQZ-X2C
#!        Role: orbital
#!     Version: 1  (Data from ccRepo)
#!----------------------------------------------------------------------
%basis
  NewGTO U

  *U basis set*

  end
```

```
  NewAuxCGTO H "autoaux" end
  NewAuxCGTO O "autoaux" end
  NewAuxCGTO U "autoaux" end
  NewAuxJKGTO H "autoaux" end
  NewAuxJKGTO O "autoaux" end
  NewAuxJKGTO U "autoaux" end
  AutoAuxLMax true
end

%coords
charge 4
coords
U -0.421989  0.032574  0.006237
O -2.038231  1.201832  1.229830
O -2.640438 -0.576352 -0.959501
O  0.383250  1.895173  1.374213
O -1.163341  1.777531 -1.419199
O  1.346727 -1.369386  0.818866
O -0.266791 -0.700521 -2.241091
O -0.792478 -2.368389 -0.236660
O  1.550335  1.027278 -0.849936
O -0.789938 -1.182696  2.137710
H -2.052076  2.077695  1.660972
H -3.080242  0.708202  1.339949
H -3.067500 -1.417594 -0.605023
H -3.376968  0.264654 -1.101881
H -1.750794 -1.450803  2.520220
H -0.126640 -1.912054  2.409525
H  0.599896 -0.541894 -2.777634
H -1.032914 -1.135441 -2.852711
H  1.381315 -2.299035  0.652120
H  2.188968 -1.285596  1.285425
H -1.496870  2.594901 -1.055482
H -1.152987  1.744988 -2.397738
H  1.499409  2.195611  1.042313
H  0.477309  1.992763  2.386995
H  1.427214  1.760383 -1.396042
H  2.369545  0.813631 -0.955901
H -0.790995 -2.978490 -1.038885
H -0.935383 -3.009113  0.528857
end
end
```

3 Excitation Energies of bare U^{4+}

Table S1: Comparison of the computed f-f transition energy values in the bare U^{4+} ion with the experimental result. M2 and M2' correspond to the same level of theory and different basis set. The Mean Absolute Error (MAE) have been computed. MAE' do not take into account the last transition.

State		DKH		X2C	
		ANO-RCC		cc-pVQZ-x2c	
J	Expt.[S1,S2]	M1	M2'	M2	M3
4	0	0	0	0	0
2	4161	4198	3987	3956	4184
5	6137	6604	6391	6448	6600
3	8984	9366	8953	8977	9347
4	9434	9713	9437	9402	9742
6	11 514	12 335	11 997	12 075	12 354
2	16 465	17 239	16 879	16 906	17 410
4	16 656	17 295	17 285	17 370	17 586
0	17 128	17 972	18 201	18 022	18 248
1	19 819	21 264	21 378	21 257	21 518
6	22 276	24 420	24 445	24 276	24 551
2	24 653	26 195	26 186	26 116	26 606
0	43 614	43 368	43 623	43 237	44 468
MAE		802	694	704	981
MAE'		852	756	733	993

4 E_0 employed for the EXAFS computation

Table S2: E_0 (eV) employed for the EXAFS computation depending on the coordination number and the experimental spectra to compare with.

System	E_0	Moll *et al.*[S3]	Hennig *et al.*[S4]	Ikeda-Ohno *et al.*[S5]	A. Uehara *et al.*[S6]
8w	MD	0	−12	−12	0
	Wigner	−2	−14	−14	2
9w	MD	1	−8	−8	5
	Wigner	0	−9	−9	3
10w	MD	3	−4	−8	6

5 Feff input for EXAFS

```
TITLE U4+ 8w
EDGE L3
 POTENTIALS
 *   ipot   z  label  l
       0    92  U     3   3
       1    8   O     3   3
*         mphase,  mpath,  mfeff,  mchi
CONTROL  0   0   0   1   1   1
PRINT      0       0       0       1
EXCHANGE 0 -4.0 0
SCF 6.0
COREHOLE RPA
TDLDA  1
CRITERIA 4.0  2.5
RPATH   6.0  ! ONLY for EXAFS
NLEG    4

S02 1.0
ATOMS
  -0.4538600   -0.0190350    0.0017400  0    U     0.4542623  0
  -2.2743710    1.2732590    1.3289730  1    O     2.9257685  0
  -2.4906550   -0.5001650   -1.0913660  1    O     2.7648883  0
   0.4985920    1.5464080    1.4248650  1    O     2.1610673  0
  -1.0211180    2.1235880   -1.1792630  1    O     2.6349515  0
   1.5619860   -1.0334880    0.7550810  1    O     2.0194170  0
  -0.2506810   -0.7415550   -2.2818600  1    O     2.4123909  0
  -0.8331130   -2.5116140   -0.1063170  1    O     2.6483175  0
   1.5903720    1.0931420   -1.0426390  1    O     2.1934764  0
  -0.8366250   -0.8558140    2.2662170  1    O     2.5628302  0
END
```

6 Feff input for XANES

```
TITLE  U4+
HOLE 4   1.0     U  L3-edge
XANES 6 0.10 0.
FMS 6.0 1
OPCONS
MPSE 2
COREHOLE RPA
*TDLDA 1
SCF 6.0 1
EXCHANGE  0 0. 0.03
RGRID 0.01
EGRID
e_grid -60 150 2

 POTENTIALS
 *   ipot   z  label  l
       0    92  U    3   3
       1    8   O    3   3
       2    1   H     2   2

*         mphase,  mpath,  mfeff,  mchi
CONTROL    1       1       1       1       1      1
PRINT      0       0       0       3

*S02 1.0
ATOMS
  -0.4689150    0.0353320    0.0298890  0    U     0.4711931  0
  -2.1185990    1.3919730    1.1024520  1    O     2.7643175  0
  -2.6142970   -0.5369170   -0.9858950  1    O     2.8451393  0
   0.6859450    1.1438090    1.5710270  1    O     2.0608118  0
  -1.1782730    1.8002170   -1.4715410  1    O     2.6066341  0
   1.6017990   -1.0195800    0.6828440  1    O     2.0178155  0
  -0.0630740   -0.8136350   -2.3106560  1    O     2.4505329  0
  -0.6733060   -2.3376620   -0.0932090  1    O     2.4344799  0
   1.3605640    1.0203090   -1.0488400  1    O     1.9980566  0
  -0.8441040   -1.0356220    2.0670850  1    O     2.4612730  0
  -2.0559280    1.9243580    1.9302110  2    H     3.4140457  0
  -3.0348420    1.1894420    1.0551420  2    H     3.4261294  0
  -2.9657370   -1.4147980   -0.7777290  2    H     3.3767013  0
  -3.1363350   -0.0172130   -1.7143190  2    H     3.5743228  0
  -1.6768710   -0.8529400    2.5863760  2    H     3.1982407  0
  -0.3963040   -1.7080890    2.6184710  2    H     3.1513513  0
   0.7306150   -0.8737130   -2.9682210  2    H     3.1792308  0
```

```
  -0.8576970   -1.5787850   -2.6989210  2    H     3.2422802  0
   1.9354360   -1.8549980    0.2285250  2    H     2.6905676  0
   2.2623290   -0.6455480    1.1739210  2    H     2.6292499  0
  -1.3495380    2.7113960   -0.9864510  2    H     3.1852797  0
  -0.8326490    2.0422920   -2.4418260  2    H     3.2904065  0
   1.1979750    1.9423910    1.3398700  2    H     2.6463708  0
   0.8848240    0.8738740    2.4652140  2    H     2.7611319  0
   1.5978070    2.0887170   -1.1432130  2    H     2.8675184  0
   2.4116340    0.8984290   -0.6947570  2    H     2.6656782  0
  -0.2287690   -2.7255870   -0.8949950  2    H     2.8778770  0
  -1.3293020   -3.0744090    0.3925020  2    H     3.3724016  0
END
```

7 Convergence of the average UV-vis spectra

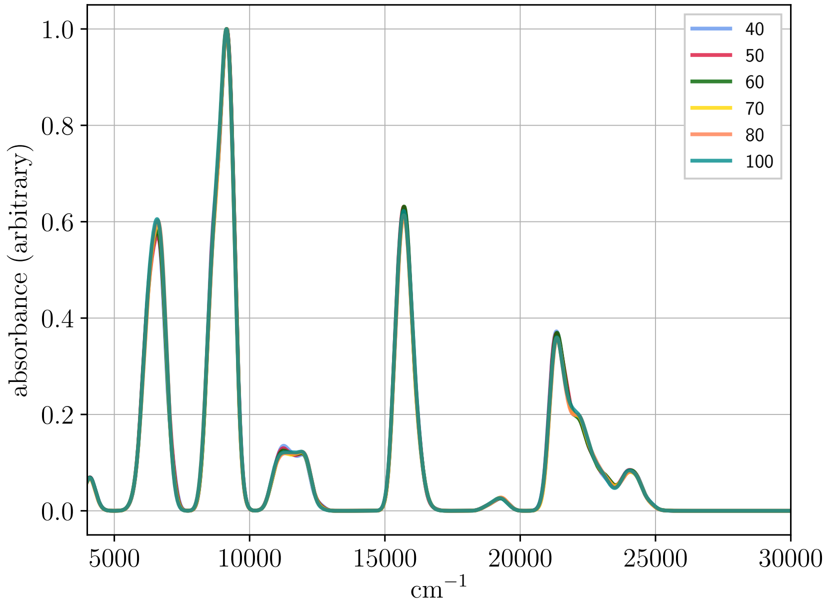

Figure S1: Average spectra of $[U(H_2O)_9]^{4+}$ from Wigner including an increasing number of individual spectra as indicated in the legend.

8 EXAFS and XANES spectra

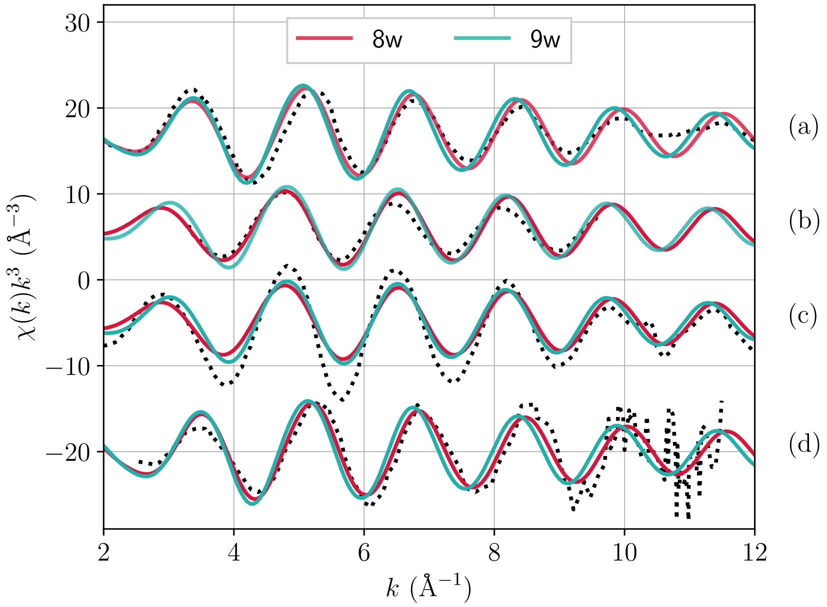

Figure S2: Average EXAFS spectra from Wigner sampling. With dotted lines, the comparison with the experimental spectrum of (a) Moll *et al.*[S3], (b) Hennig *et al.*[S4], (c) Ikeda-Ohno *et al.*[S5] and (d) A. Uehara *et al.*[S6], which have arbitrary intensity.

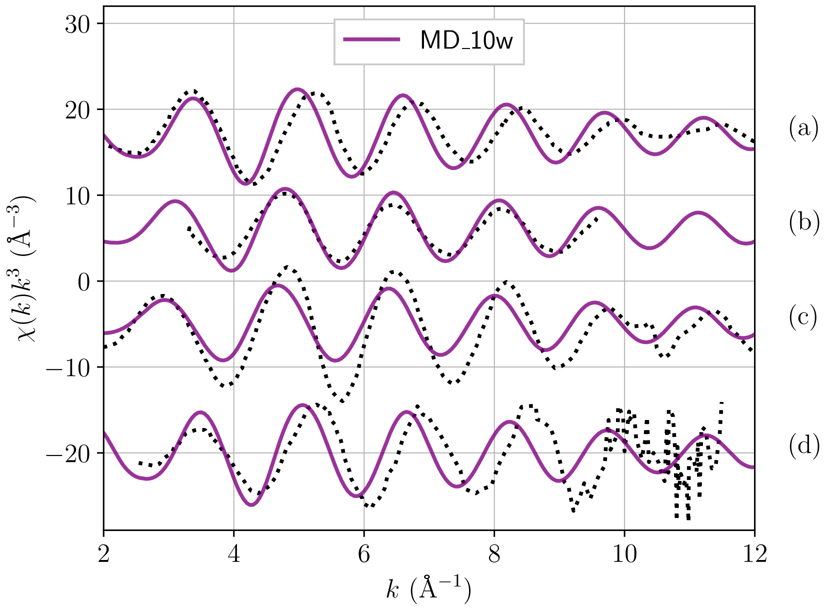

Figure S3: Average EXAFS spectra for $[U(H_2O)_{10}]^{4+}$ from MD sampling. From top to bottom, the comparison with the experimental spectrum of Moll *et al.*[S3], Hennig *et al.*[S4], Ikeda-Ohno *et al.*[S5] and A. Uehara *et al.*[S6], which have arbitrary intensity.

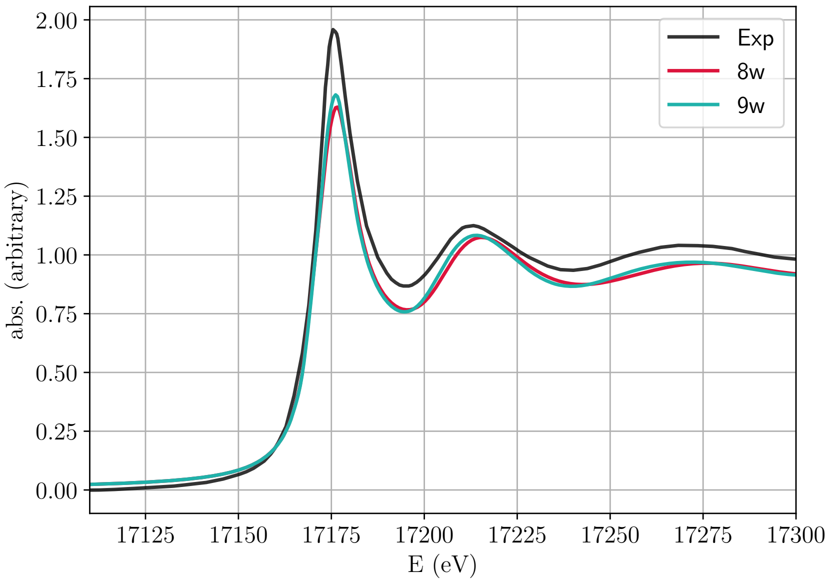

Figure S4: Comparison of the average XANES spectra from Wigner sampling with the experimental one recorded by Ikeda-Ohno *et al.*[S5].

9 Average UV-vis spectra computed with M1 method in wavelength

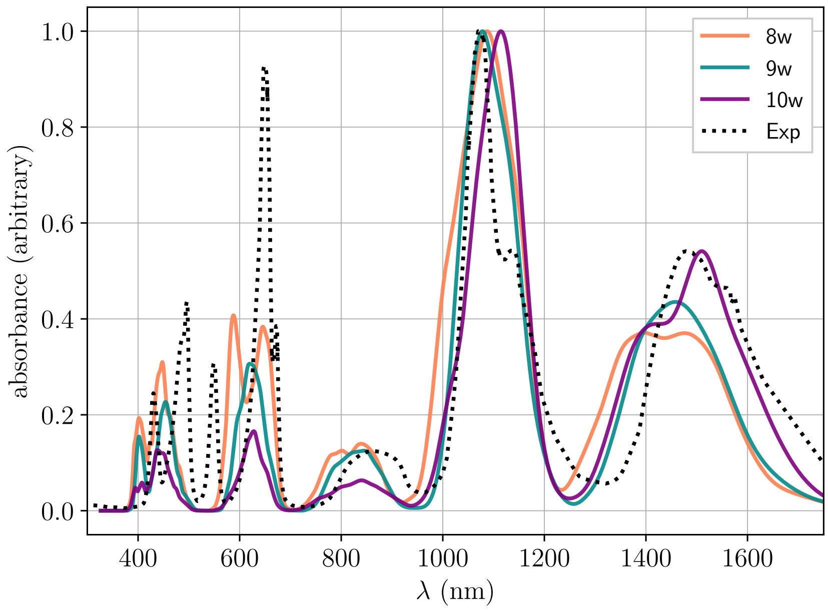

(a) MD simulations

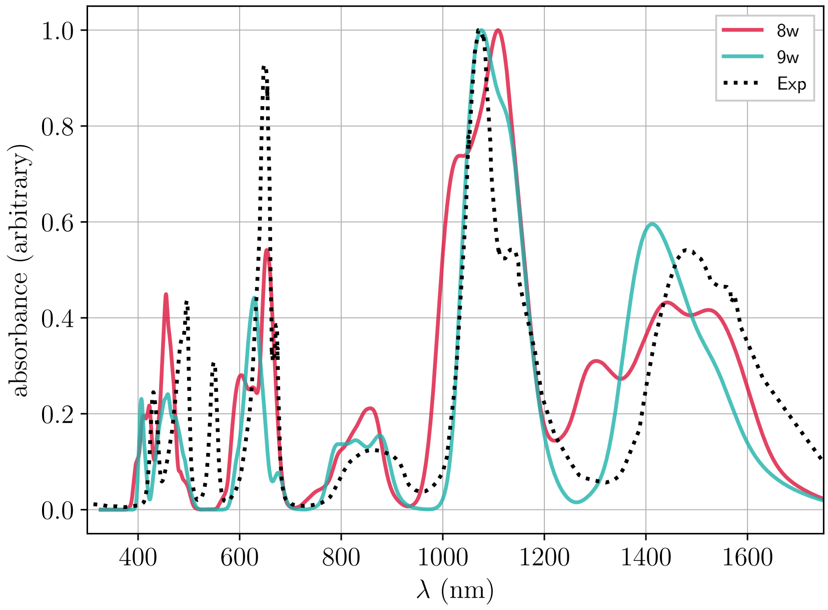

(b) Wigner distribution

Figure S5: Average spectra represented in wavelength for $[U(H_2O)_n]^{4+}$ (a) from MD simulation with n=8, 9, 10, and (b) from Wigner distribution with n=8, 9, with the M1 method.

10 Average UV-vis spectra computed with M1 method without normalisation

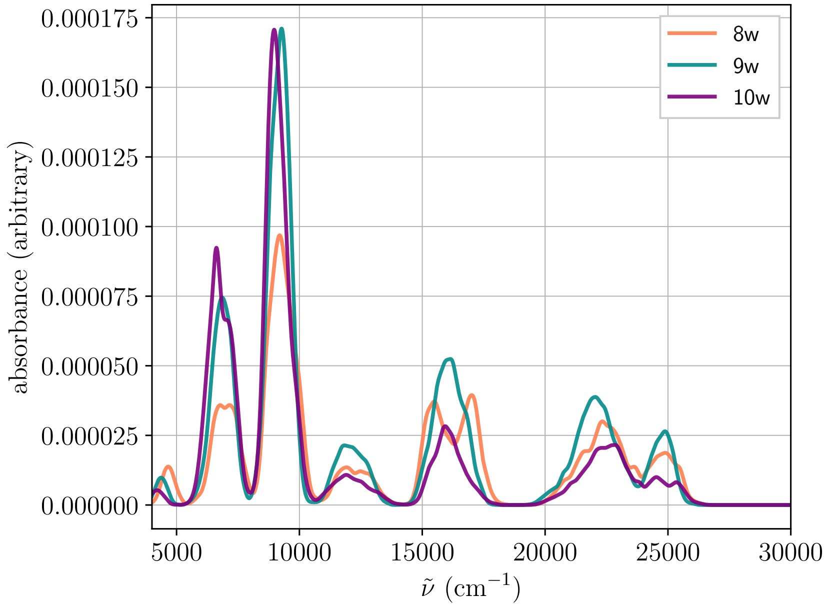

(a) MD simulations

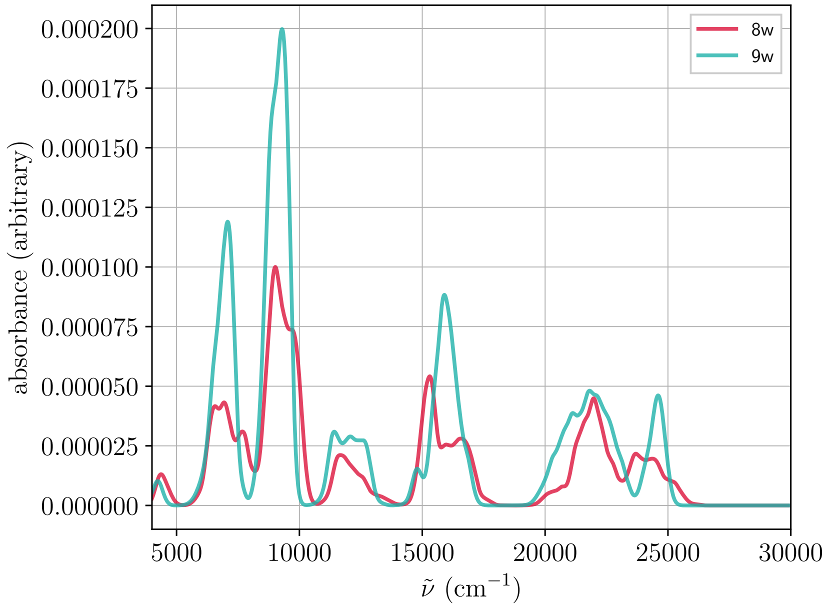

(b) Wigner distribution

Figure S6: Average spectra without normalisation for $[U(H_2O)_n]^{4+}$ (a) from MD simulation with n=8, 9, 10, and (b) from Wigner distribution with n=8, 9, with the M1 method.

11 Average UV-vis spectra computed with M2 method

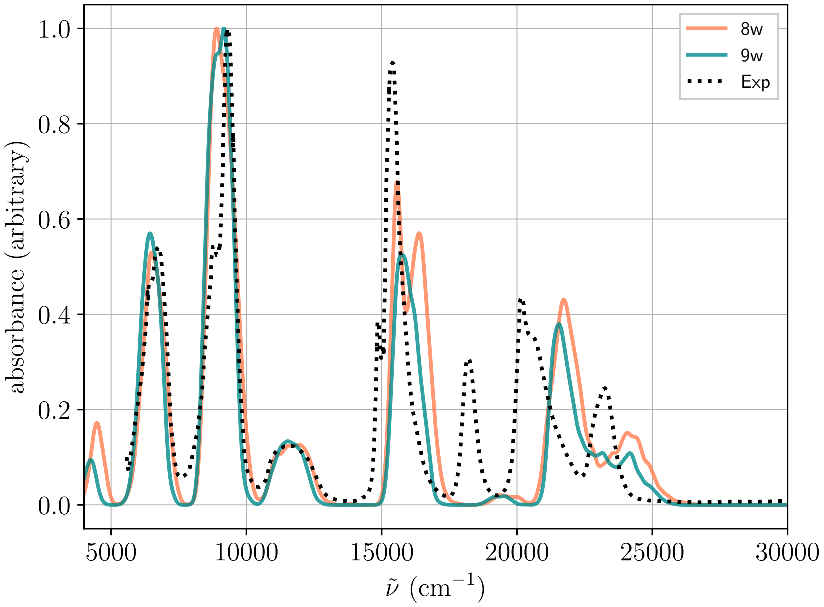

Figure S7: Average spectrum from MD sampling for $[U(H_2O)_n]^{4+}$ with n=8, 9 obtained by the M2 method.

12 Average UV-vis spectra computed with M2 method in wavelength

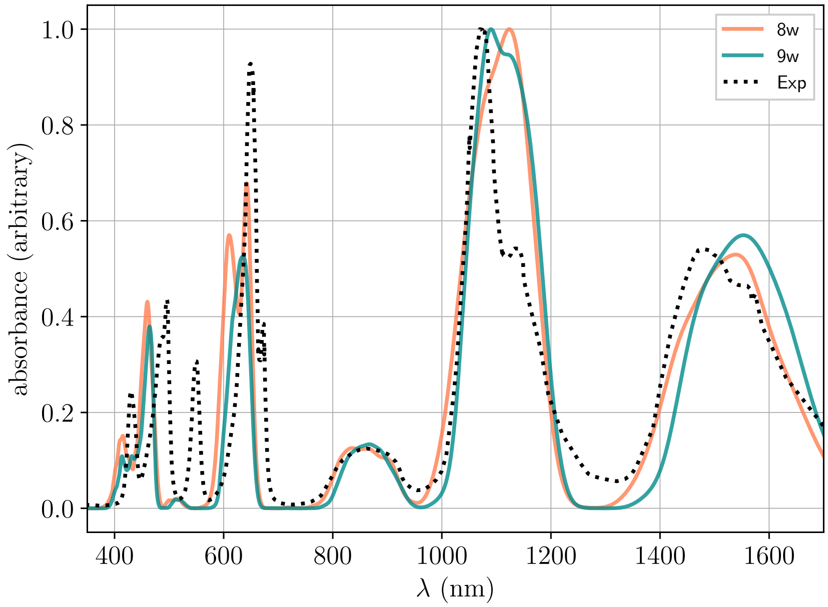

(a) MD simulations

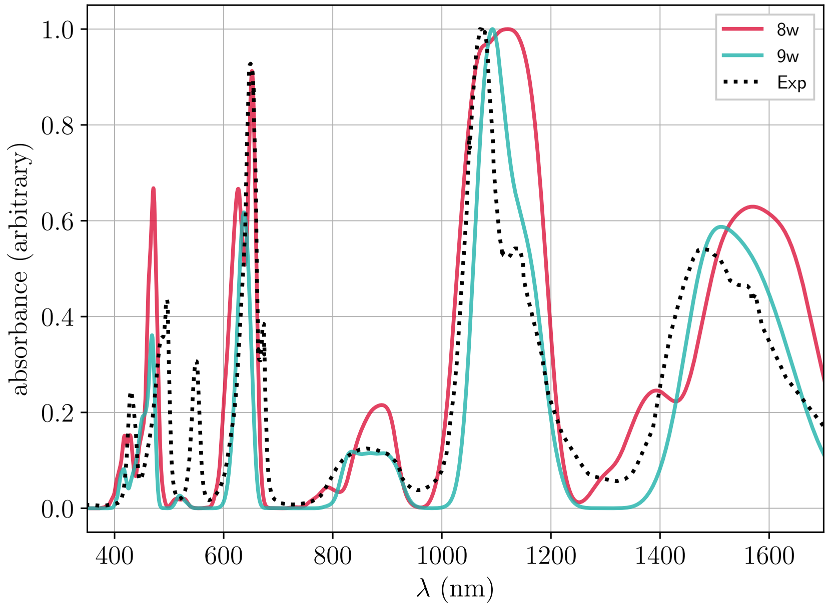

(b) Wigner distribution

Figure S8: Average spectra represented in wavelength for $[U(H_2O)_n]^{4+}$ (a) from MD simulation with n=8, 9, 10, and (b) from Wigner distribution with n=8, 9, with the M2 method.

13 Average UV-vis spectra computed with M3 method

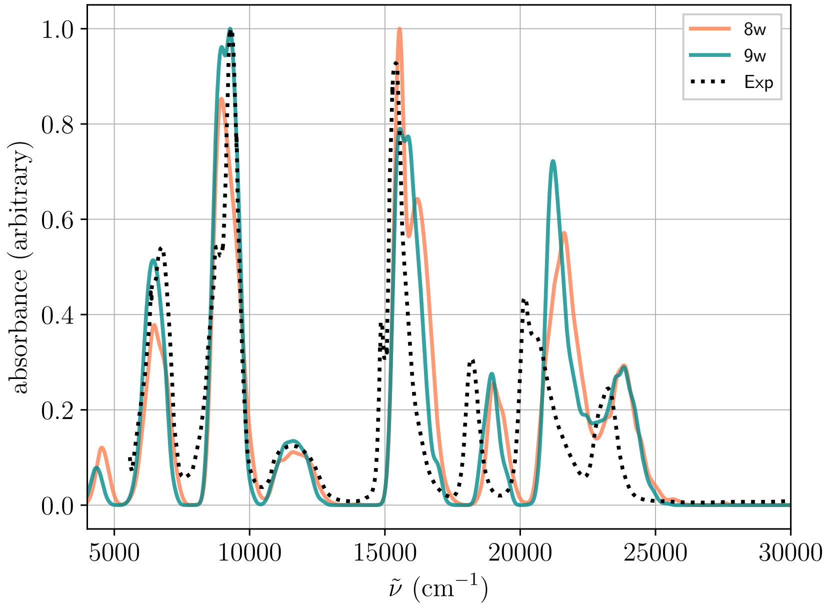

Figure S9: Average spectrum from MD sampling for $[U(H_2O)_8]^{4+}$ and $[U(H_2O)_9]^{4+}$ obtained by the M3 method.

14 Average UV-vis spectra computed with M3 method in wavelength

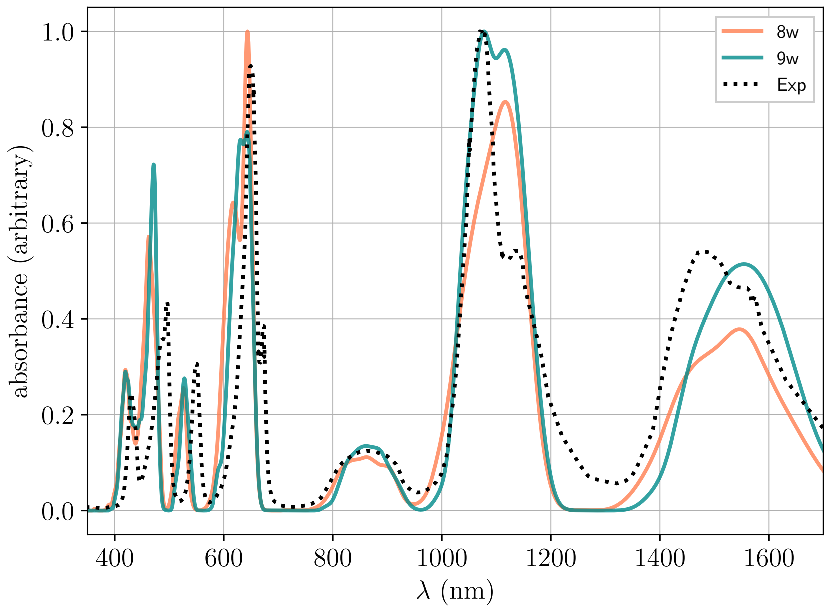

(a) MD simulations

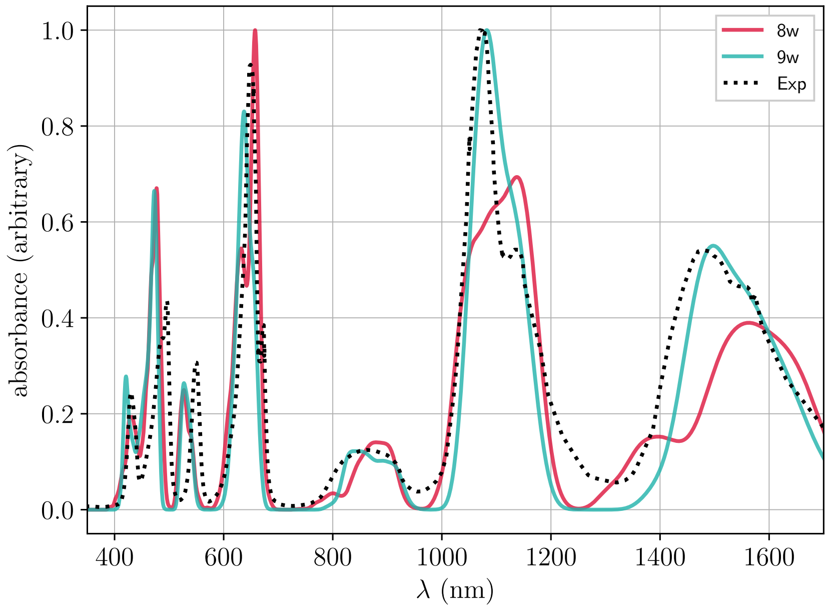

(b) Wigner distribution

Figure S10: Average spectra represented in wavelength for $[U(H_2O)_n]^{4+}$ (a) from MD simulation with n=8, 9, 10, and (b) from Wigner distribution with n=8, 9, with the M3 method.

15 Speciation as a function of pH

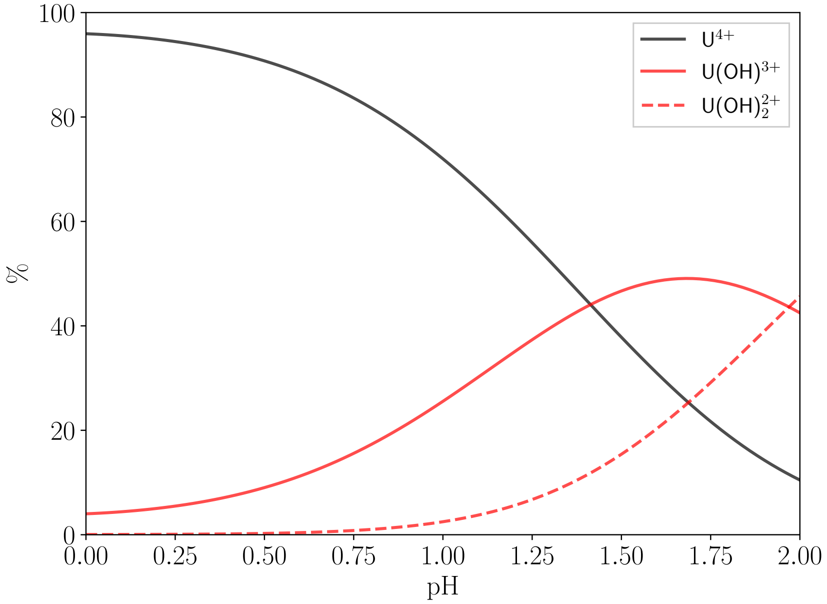

Figure S11: Speciation diagram as a function of pH using the thermodynamic data of Yan *et al.*[S7].

Table S3: Percentages of the species U^{4+}, $U(OH)^{3+}$ and $U(OH)_2^{2+}$ employed for the reconstruction of the theoretical spectra as a function of pH, following the speciation diagram contructed from the thermodynamic data of Yan *et al.*[S7] and the one published in the work of Lehmann *et al.*[S8]

pH	$\%[U(H_2O)_9]^{4+}$	$\%[U(OH)]^{3+}$	$\%[U(OH)_2]^{2+}$
Yan *et al.*[S7]			
0.7	81	16	3
1.0	60	30	10
1.3	36	38	26
1.7	11	27	62
2.0	3	17	80
Lehmann *et al.*[S8]			
0.7	86	14	0
1.0	72	25	3
1.3	52	40	8
1.7	25	49	24
2.0	10	44	46

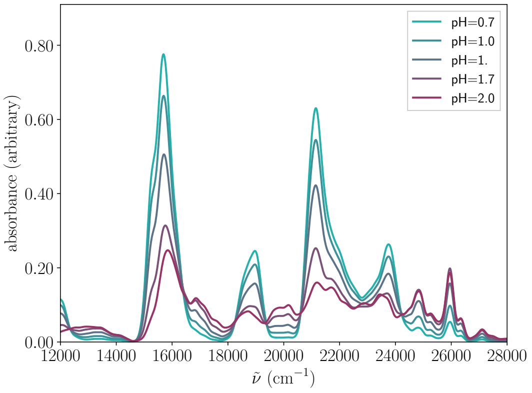

(a)

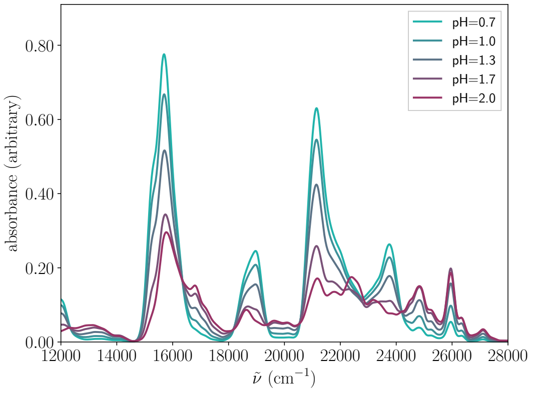

(b)

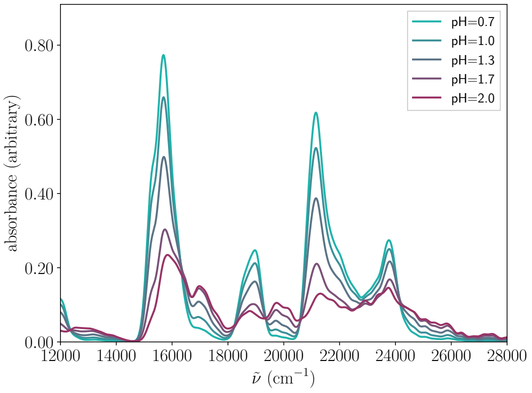

(c)

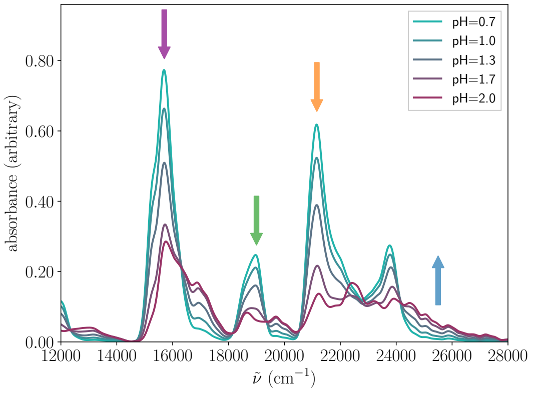

(d)

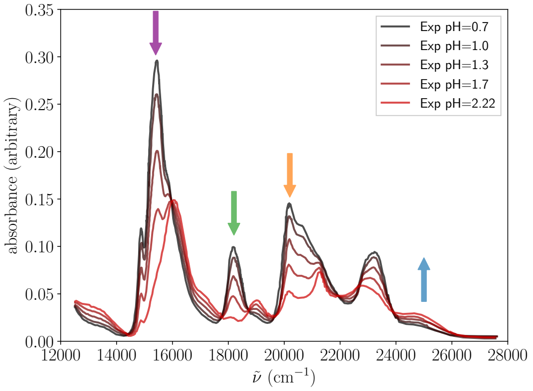

(e) Experimental spectra

Figure S12: Weighted sums of $[U(H_2O)_9]^{4+}$ and $[U(OH)_m(H_2O)_n]^{4-m+}$ Wigner-generated spectra, being m=1,2 and n=5-7, to show the effect of the percentage of hydrolysis: (a)$[U(H_2O)_9]^{4+}$ and $[U(OH)_2(H_2O)_5]^{2+}$ and $[U(OH)(H_2O)_6]^{3+}$; (b)$[U(H_2O)_9]^{4+}$ and $[U(OH)_2(H_2O)_6]^{2+}$ and $[U(OH)(H_2O)_6]^{3+}$; (c)$[U(H_2O)_9]^{4+}$ and $[U(OH)_2(H_2O)_5]^{2+}$ and $[U(OH)(H_2O)_7]^{3+}$; (d)$[U(H_2O)_9]^{4+}$ and $[U(OH)_2(H_2O)_6]^{2+}$ and $[U(OH)(H_2O)_7]^{3+}$. The percentages employed are those obtained with the thermodynamic data published in the work of Yan *et al.*[S7], summarized in Table S3.

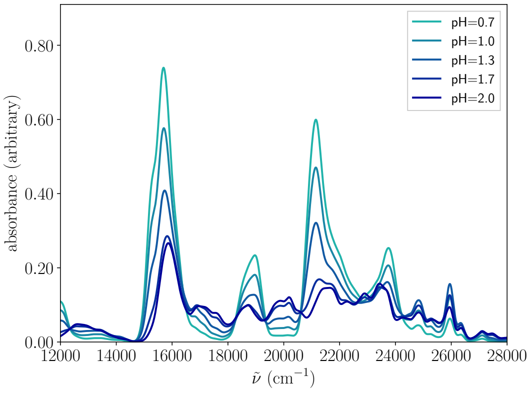

(a)

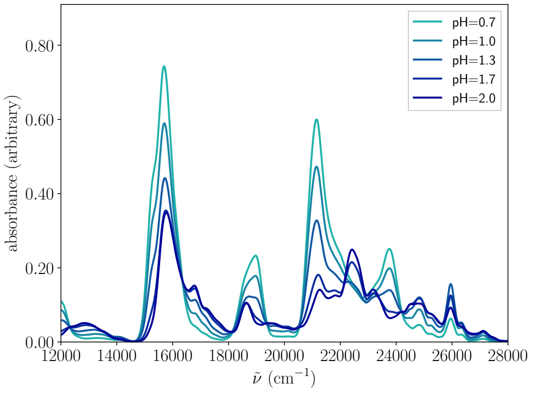

(b)

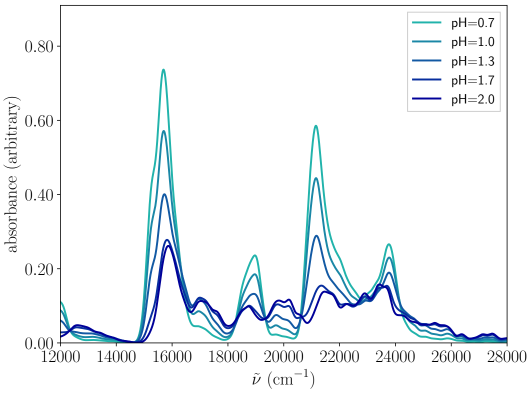

(c)

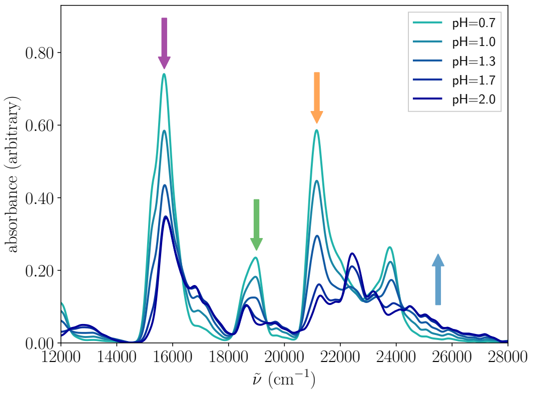

(d)

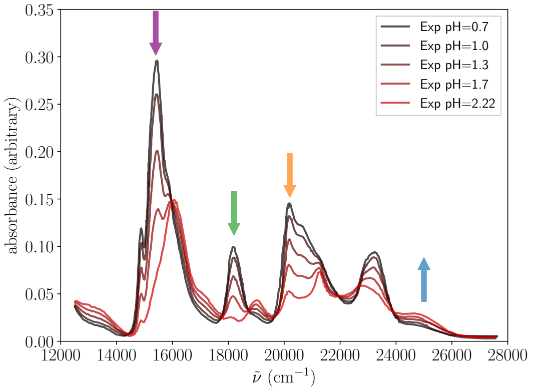

(e) Experimental spectra

Figure S13: Weighted sum of $[U(H_2O)_9]^{4+}$ and $[U(OH)_m(H_2O)_n]^{4-m+}$ Wigner-generated spectra, being m=1,2 and n=5-7, to show the effect of the percentage of hydrolysis: (a)$[U(H_2O)_9]^{4+}$ and $[U(OH)_2(H_2O)_5]^{2+}$ and $[U(OH)(H_2O)_6]^{3+}$; (b)$[U(H_2O)_9]^{4+}$ and $[U(OH)_2(H_2O)_6]^{2+}$ and $[U(OH)(H_2O)_6]^{3+}$; (c)$[U(H_2O)_9]^{4+}$ and $[U(OH)_2(H_2O)_5]^{2+}$ and $[U(OH)(H_2O)_7]^{3+}$; (d)$[U(H_2O)_9]^{4+}$ and $[U(OH)_2(H_2O)_6]^{2+}$ and $[U(OH)(H_2O)_7]^{3+}$. The percentages employed are those published in the work of Lehmann *et al.*[S8] and summarized in Table S3.

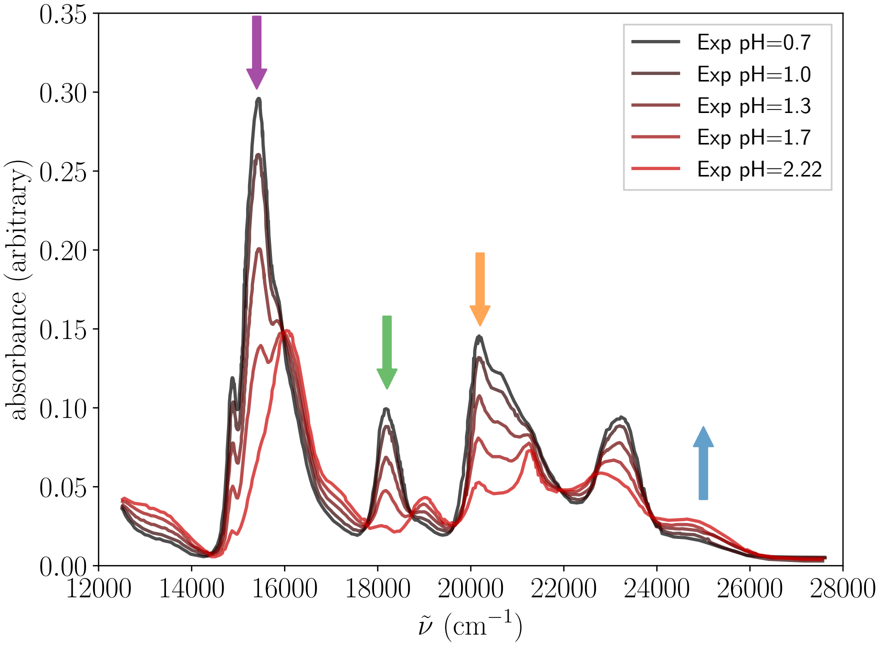

(a)

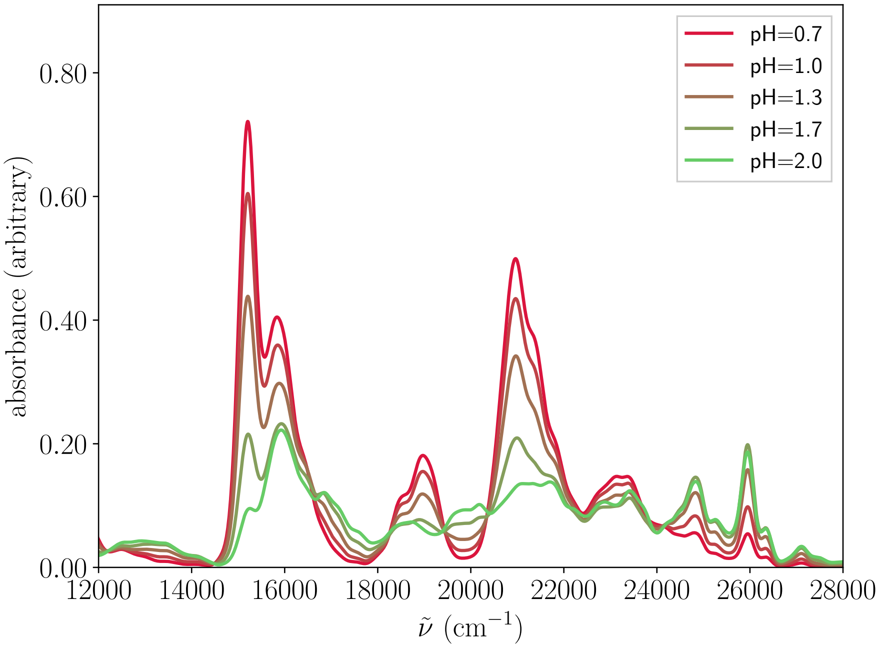

(b)

Figure S14: a) Experimental spectra measured by Cha *et al.*[S9] as a function of pH. b) Weighted sum of $[U(H_2O)_8]^{4+}$, $[U(OH)(H_2O)_6]^{3+}$ and $[U(OH)_2(H_2O)_5]^{2+}$ Wigner-generated spectra to show the effect of the percentage of hydrolysis. The percentages employed are summarized in Table S3, where the data is limited to pH=2.0.

References

(S1) Wyart, J. F.; Kaufman, V.; Sugar, J. Analysis of the Spectrum of Four-Times-Ionized Uranium (U5). *Phys. Scr.* **1980**, *22*, 389, DOI: 10.1088/0031-8949/22/4/011.

(S2) Deurzen, C. H. H. V.; Rajnak, K.; Conway, J. G. Uranium Five (U v), the $^1S_0$ Level, and a Parametric Analysis of the $5f^2$ Configuration. *J. Opt. Soc. Am. B: Opt. Phys.* **1984**, *1*, 45–47, DOI: 10.1364/JOSAB.1.000045.

(S3) Moll, H.; Denecke, M. A.; Jalilehvand, F.; Sandström, M.; Grenthe, I. Structure of the Aqua Ions and Fluoride Complexes of Uranium(IV) and Thorium(IV) in Aqueous Solution an EXAFS Study. *Inorg. Chem.* **1999**, *38*, 1795–1799, DOI: 10.1021/ic981362z.

(S4) Hennig, C.; Tutschku, J.; Rossberg, A.; Bernhard, G.; Scheinost, A. C. Comparative EXAFS Investigation of Uranium(VI) and -(IV) Aquo Chloro Complexes in Solution Using a Newly Developed Spectroelectrochemical Cell. *Inorg. Chem.* **2005**, *44*, 6655–6661, DOI: 10.1021/ic048422n.

(S5) Ikeda-Ohno, A.; Hennig, C.; Tsushima, S.; Scheinost, A. C.; Bernhard, G.; Yaita, T. Speciation and Structural Study of U(IV) and -(VI) in Perchloric and Nitric Acid Solutions. *Inorg. Chem.* **2009**, *48*, 7201–7210, DOI: 10.1021/ic9004467.

(S6) A. Uehara; Fujii, T.; Matsuura, H.; Sato, N.; Nagai, T.; Minato, K.; Yamana, H.; Okamoto, Y. EXAFS Analysis of Uranium(IV) and Thorium(IV) Complexes in Concentrated $CaCl_2$ Solutions. *Proc. Radiochim. Acta* **2011**, *1*, 161–165, DOI: 10.1524/rcpr.2011.0030.

(S7) Yan, Y.; Cevirim-Papaioannou, N.; Gaona, X.; Fellhauer, D.; Altmaier, M. Thermodynamic Description of U(IV) Solubility and Hydrolysis in Chloride Systems: Pitzer Activity Model for the System U^{4+}–Na^{+}–Mg^{2+}–Ca^{2+}–H^{+}–Cl^{-}–OH^{-}–$H_2O(l)$. *Appl. Geochem.* **2024**, 106091, DOI: 10.1016/j.apgeochem.2024.106091.

(S8) Lehmann, S.; Foerstendorf, H.; Zimmermann, T.; Patzschke, M.; Bok, F.; Brendler, V.; Stumpf, T.; Steudtner, R. Thermodynamic and Structural Aspects of the Aqueous Uranium(IV) System – Hydrolysis vs. Sulfate Complexation. *Dalton Trans.* **2019**, *48*, 17898–17907, DOI: 10.1039/C9DT02886B.

(S9) Cha, W.; Kim, H.-K.; Cho, H.; Cho, H.-R.; Jung, E. C.; Lee, S. Y. Studies of Aqueous U(IV) Equilibrium and Nanoparticle Formation Kinetics Using Spectrophotometric Reaction Modeling Analysis. *RSC Adv.* **2020**, *10*, 36723–36733, DOI: 10.1039/D0RA05352J.
